# Limited Agreement of Independent RNAi Screens for Virus-Required Host Genes Owes More to False-Negative than False-Positive Factors

**DOI:** 10.1371/journal.pcbi.1003235

**Published:** 2013-09-19

**Authors:** Linhui Hao, Qiuling He, Zhishi Wang, Mark Craven, Michael A. Newton, Paul Ahlquist

**Affiliations:** 1 Institute of Molecular Virology, University of Wisconsin-Madison, Madison, Wisconsin, United States of America; 2 Howard Hughes Medical Institute, University of Wisconsin-Madison, Madison, Wisconsin, United States of America; 3 Department of Statistics, University of Wisconsin-Madison, Madison, Wisconsin, United States of America; 4 Department of Biostatistics and Medical Informatics, University of Wisconsin-Madison, Madison, Wisconsin, United States of America; 5 Department of Computer Sciences, University of Wisconsin-Madison, Madison, Wisconsin, United States of America; 6 Morgridge Institute for Research, Madison, Wisconsin, United States of America; University of Illinois at Urbana-Champaign, United States of America

## Abstract

Systematic, genome-wide RNA interference (RNAi) analysis is a powerful approach to identify gene functions that support or modulate selected biological processes. An emerging challenge shared with some other genome-wide approaches is that independent RNAi studies often show limited agreement in their lists of implicated genes. To better understand this, we analyzed four genome-wide RNAi studies that identified host genes involved in influenza virus replication. These studies collectively identified and validated the roles of 614 cell genes, but pair-wise overlap among the four gene lists was only 3% to 15% (average 6.7%). However, a number of functional categories were overrepresented in multiple studies. The pair-wise overlap of these enriched-category lists was high, ∼19%, implying more agreement among studies than apparent at the gene level. Probing this further, we found that the gene lists implicated by independent studies were highly connected in interacting networks by independent functional measures such as protein-protein interactions, at rates significantly higher than predicted by chance. We also developed a general, model-based approach to gauge the effects of false-positive and false-negative factors and to estimate, from a limited number of studies, the total number of genes involved in a process. For influenza virus replication, this novel statistical approach estimates the total number of cell genes involved to be ∼2,800. This and multiple other aspects of our experimental and computational results imply that, when following good quality control practices, the low overlap between studies is primarily due to false negatives rather than false-positive gene identifications. These results and methods have implications for and applications to multiple forms of genome-wide analysis.

## Author Summary

Genome-wide RNA interference assays of gene functions offer the potential for systematic, global analysis of biological processes. A pressing challenge is to develop meta-analysis methods that effectively combine information from multiple studies. One puzzle is that implicated gene lists from independent studies of the same process often show relatively low overlap. This disagreement might arise from false-positive factors, such as imperfect gene targeting (off-target effects), or from false negatives if separate studies access different components of large, complex systems. We present new methods to examine the relations between individual genome-wide RNAi studies, using studies of host genes in influenza virus replication as a test case. We find that cross-study agreement is greater than suggested by overlap of reported gene lists. This better agreement is evidenced by the strong relation of independent gene lists in functional pathways and protein interaction networks, and by a statistical model that relates multi-study, gene-level findings to factors driving correct, false-negative, and false-positive gene identification. Our analysis of multiple genome-wide studies predicts that there are many undetected host genes important for influenza virus infection, and that false negatives are the major concerns for genome-wide studies.

## Introduction

RNA interference (RNAi) is a gene-specific silencing process directed by short double stranded RNAs or small interfering RNAs (siRNAs) that can “knock down” expression of a selected gene by inducing messenger RNA (mRNA) degradation in a sequence-specific manner [1]. RNAi has been widely used as a molecular tool to selectively inhibit the expression of a chosen gene. By expanding this technique to use large-scale RNAi libraries, high-throughput RNAi analysis has become a powerful approach to screen essentially all genes of an organism, to identify gene functions that support or modulate any biological process of interest.

Genome-wide RNAi analyses have been used to study many important biological processes and have provided novel, key insights. One important application of genome-wide RNAi screening has been to identify host genes that are required for the replication of a particular virus [2]–[4]. In several cases, two or more independent RNAi screens have been performed to identify host factors required by the same viruses [5]–[11]. An emerging challenge shared with some other genome-wide approaches is that such independent genome-wide RNAi studies often exhibit limited overlap in the lists of genes implicated. For example, there is only 3–6% overlap of gene lists identified from three genome wide RNAi studies for host factors of HIV [6], [7], [12], [13].

Lack of overlap between studies must be due to some combination of false-positive and false-negative factors. If the dissimilarity between the sets of identified host factors is predominantly from false-positive factors, the majority of the genes identified from a genome wide RNAi study would be false positives. In this case, the number of genes involved in a biological process such as virus infection would be low relative to the number implicated. On the other hand, a high false-negative rate could also induce low agreement, since in this case each study would confirm just a small subset of involved genes, and many more genes than one study could identify might be implicated in the process.

A critical step to reduce false discoveries is good quality control, which is essential due to the limited number of repeats possible for genome-wide analyses, frequently high assay noise, the potential for systematic errors, and other effects. Relevant quality control practices include adequately designed positive and negative controls, proper patterning of samples and controls on microtiter assay plates, data display approaches suitable for revealing systematic errors, etc. Nevertheless, even good quality control cannot eliminate error, so that quality control is an essential but not sufficient basis for effective RNAi analysis. Appropriate data analysis, such as false discovery rate (FDR) controlling procedures, should also help to minimize false discoveries [14]. However, the FDR is controlled within each study, and is not designed to control false-positive error rates caused by sources of variation between studies. Moreover, an inherent problem for large-scale dataset processing is that a strict cut-off to limit false-positive results increases the number of false negative results, and vice versa [1]. Depending on the goal of a study, the tolerance for false positives or false negatives might be adjusted accordingly to generate the final results.

Of course, data analysis is not the sole source or solution for false-positive and-negative results. Both technical and biological sources also contribute to false discoveries and non-discoveries in genome-wide RNAi studies. An siRNA may silence one or more genes besides the targeted one [15], owing to incomplete sensitivity and specificity of siRNAs [16]–[19]. Beyond these off-target effects, another false-positive factor is measurement error intrinsic to the complex phenotypic readouts typically used. Similarly, many experimental issues contribute to false negatives. For example, the organism under study may have genetic redundancies that limit the accessibility of certain functions to phenotypic manipulation by knocking down a single gene [20], [21]. Furthermore, genes with undetectable expression or whose knock down results in cytotoxicity are usually excluded from such RNAi analyses, further reducing the number of genes that are accessed in such “genome-wide” studies [22]. Besides the above issues, false negatives also can be generated by systematic errors such as plate position effects [23], by failure to control for variation in local cell environments [24], [25], by inefficiencies in knocking down targeted genes [26], and by other effects. For these and other reasons noted above, use of well-chosen quality control as well as analytic methods are critical for producing high quality screening results [23], [27].

**Figure 1:**
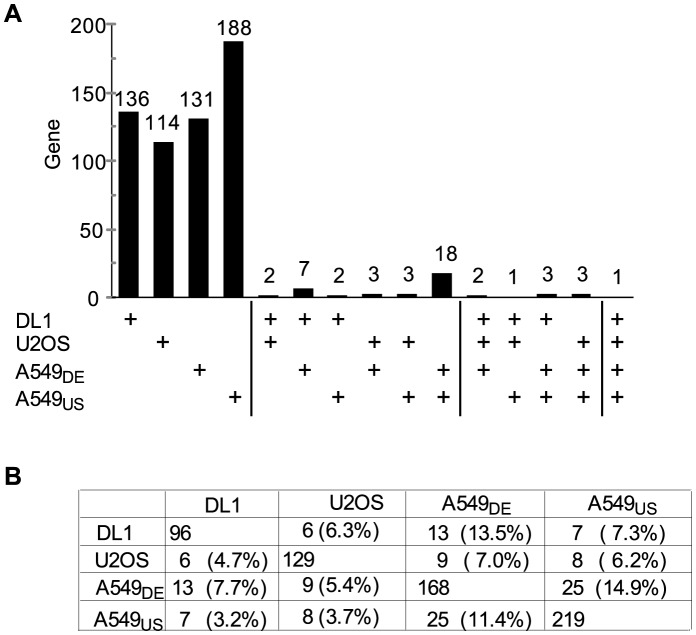
Distribution across four RNAi studies of genes implicated in influenza virus replication. **A**) Distribution of genes confirmed by the four RNAi studies designated as DL1, U2OS, A549_US_ and A549_DE_ searching for host factors of influenza virus. The bars show the counts of genes confirmed in any 1, 2, 3 or 4 studies (indicated by + in table below the graph). Note that most genes were only confirmed in one study. **B**) Pairwise overlap of confirmed genes in the four RNAi studies. The table shows the number of genes confirmed by each study (main diagonal) and the pairwise overlap between any two indicated studies (flanking cells), as both the absolute number of genes and as a percentage (in parentheses) of the total genes confirmed in the study of the relevant column. Fisher exact p-values are less than 10∧(−10) for all pair-wise comparisons.

To better understand RNAi screening and the relative contributions of false positives and false negatives, we performed a meta-analysis of four recent studies to identify host genes involved in the replication of influenza virus, an important human pathogen [8]–[11], [28]. Despite differences in the RNAi libraries and cell lines used, these studies, including one from our laboratory [9], employed similar two-step approaches. All studies began with a high-throughput primary screen with an RNAi library targeting the whole genome. Candidate genes from this primary screen then were re-tested for function in virus replication in repeated secondary validation assays with individual siRNAs. Similar to prior HIV results, the pair-wise overlaps among the confirmed gene lists were only 3–15% (Figure 1).

Using a multi-faceted meta-analysis, we found that the independent gene lists are strongly interlinked by functional pathways and protein-protein interactions, and that their low overlap is due primarily to false negative rather than false-positive factors. First, there is substantially higher agreement between studies from the perspective of functional categories rather than gene lists. Second, the combined list of genes confirmed by all studies embodies a much richer network of molecular interactions than expected by chance. Thus, genes identified in independent RNAi studies are physically as well as functionally connected. Finally, we developed a new statistical model that incorporates the main experimental features of RNAi screening and that, upon fitting to the data via likelihood and Bayesian techniques, estimates the major intrinsic parameters governing false positives and false negatives. This model well duplicates the statistical patterns in the multi-study gene level data, and indicates that low overlap arises primarily from false negative factors. Thus, our bioinformatic and statistical analyses show that current genome-wide RNAi screens each reveal a highly useful but partial glimpse of a larger whole.

## Methods

### Data collection and preliminary analysis

Gene-level results from primary and secondary screens were collected from four genome-wide RNAi screens to identify host factors crucial for influenza virus replication [8]–[11]. All human genes were mapped to Entrez IDs (NCBI, http://www.ncbi.nlm.nih.gov/); human orthologs for *Drosophila* genes were extracted from www.ensembl.org. Table S1 lists for each study the Entrez IDs of all confirmed genes, followed by unconfirmed primary candidates. Study-specific lists of confirmed genes were compared using a mean overlap fraction (MOF). On average over all pairings of lists, MOF is the mean value of the proportion of one list that overlaps with another.

### Gene-set analysis

Gene-set analysis was performed using confirmed genes from each of the four studies and functional-category information from the Gene Ontology (GO) project, as accessed using the R and Bioconductor systems (www.bioconductor.org). We applied the recently-developed multi-function analyzer (MFA) [29], [30] to process each gene list (four study-specific gene lists and one combined list). Briefly, MFA provides a model-based analysis of a gene list. A gene's presence on the list is explained by latent binary activities of GO terms which annotate it; through model-based computations MFA determines the posterior activity probabilities over all terms in the collection. It refines model-based gene set analysis (MGSA) [31] by encoding natural constraints on the function-level activities in order to improve statistical efficiency. Like MGSA, MFA addresses deficiencies of simpler gene-set methods (e.g., in handling set overlaps) by the simultaneous analysis of all sets in a collection. Gene-permutation was used to calibrate the between-study agreement of MFA-derived set lists. Specifically, data were organized in an incidence matrix with rows for genes and columns for studies, and containing indicators of confirmation. Permutation proceeded by shuffling row labels (gene IDs) and retaining the numbers of genes co-confirmed in two, three, and four studies, and expressed the null assumption of no functional association between studies. We computed Monte Carlo p-values using the MFA results from the observed data and from 999 permuted versions. Computations used *GO.db* version 2.8.0; and *org.Hs.eg.db* version 2.8.0 (September, 2012). In total 2472 GO terms were used; these are all terms that have non-empty intersection with the combined list of influenza-related genes and that are moderate in size (between 5 and 50 human genes). The size constraint improved computational efficiency without much loss in functional information.

### Network analysis

Analysis of molecular interactions among the confirmed genes was performed in order to further assess the relatedness of the genes, both within studies and across studies. We assembled *interaction graphs*; these are data structures in which the vertices correspond to genes and the edges correspond to protein-protein interactions. The set of interactions used consisted of 47,647 human, protein-protein interactions from the BioGRID database [9].

A *connected component* is a maximal subgraph in which any two genes in the subgraph are connected by one or more paths. To assess the extent of inter-relatedness within a list of confirmed genes, we considered several properties of the subgraph that results from selecting only the confirmed genes and their impinging interactions. First, we measured the number of edges in the resulting connected components and the size (in terms of genes) of the largest connected component. Second, we measured the *average degree* of the vertices in the confirmed-gene subgraph. The degree of a vertex in a graph is the number of other vertices to which it has edges. Thus we measured, on average, the number of other confirmed genes with which each confirmed gene has known interactions.

To determine if any of these measures is surprisingly large, we employed a Monte Carlo test in which the null hypothesis is that the measure can be accounted for by a randomly selected set of pseudo “confirmed” lists. Given a set of *n* actual confirmed genes, this test involved repeated random selection of gene sets of size *n*. We selected these random gene sets such that the degree of connectivity of the selected genes, with respect to the entire interaction graph, was the same as the degree of connectivity of the given list of confirmed genes. That is, if there are three genes in the confirmed list that each has eight known interactions with other genes in the genome, then our randomly selected gene sets would also include exactly three genes with eight interactions each. Note that the degree of connectivity we consider when doing this selection process refers to the connectivity of a given gene to *all* genes in the genome, as opposed to its connectivity to other confirmed genes. For the results reported here, our Monte Carlo p-values involved 9,999 iterations.

To determine if there were more relationships between pairs of genes confirmed in different studies than would be expected by chance, we pooled the confirmed genes from the four independent studies and determined the connected components that resulted from this set of pooled genes. As before, we counted the number of edges in connected components, determined the size of the largest connected component, determined the average degree for confirmed genes, and assessed the statistical significance of each measure using a Monte Carlo methodology. Additionally, to assess the extent to which the genes confirmed in separate studies were related, we counted the number of *spanning edges* in the connected components. A spanning edge is one that represents an interaction between two genes that were confirmed in different studies. We used a Monte Carlo test to measure the statistical significance of the number of spanning edges we observed. Each iteration of the Monte Carlo test involved randomly selecting, for each study in the pool, a set of pseudo “confirmed” genes which have the same degree of connectivity as the actual confirmed genes in that study. Given these randomly chosen sets, we counted the number of spanning edges in each as we did with the actual data. Again, our Monte Carlo p-values used 9,999 iterations.

### Model-based statistical analysis

To provide reasoned inferences about factors affecting among-study gene-level agreement, we developed a statistical model for genome-wide RNAi studies and corresponding likelihood-based analysis methods. The model formulates relationships among: system-level parameters that affect sensitivity and various error rates, gene-level and study-level latent variables that transduce information about the system to information at the gene-level, and gene-level, multi-study data on both detection and confirmation by RNAi screening. In its generative form, the model specifies the probability of observing any particular multi-study data set. In its inferential form, it indicates the likelihood assigned to any particular parameter setting in light of observed data [32].

#### Multinomial backbone

For each study 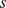 in the set of four studies, and each gene 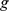 in the human genome, we introduce 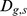 to indicate whether or not (1 or 0) gene 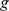 was *detected* in the primary screen of study 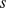, and similarly 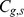 to indicate whether or not 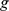 was *confirmed* in the corresponding secondary screen. Multi-study genome-wide data are thereby reduced to a tabular form, in which we count the number 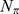 of genes having pattern π in detection/confirmation across the four studies (see table with multi-study data in count format below). For example, exactly 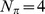 genes were detected and confirmed in study *DL1*, detected but not confirmed in *U2OS*, and not detected in the other two studies. Overall, there are 81 possible multi-study detection and confirmation patterns across the four studies, as summarized below in a table with the associated counts for each pattern. In our model, the data vector 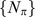 is considered to have a multinomial distribution in which pattern probabilities 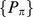 are determined by parameters governing the system, as induced through the following probability model.

#### Involvement

Whether or not a gene 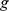 is truly *involved* in influenza-virus replication is unknown *a priori*, and this fact is expressed by the latent binary variable 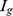. In some cell type, an error-free measurement of a true knockdown, in the absence of off-target effects, would show a phenotype if and only if 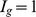. The parameter 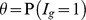 is the genome-wide rate (*i.e.*, probability) of true involvement. Fixing the genome size at 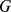, the number of truly involved genes is 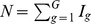, which has expected value 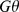. The distribution of gene-level data depends on 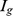 through additional factors expressing sources of variation that affect knockdown and phenotype, as discussed in the following sections.

#### Accessibility

A variety of factors could block either the knockdown of a gene or the phenotype of a knocked-down gene. The gene may not be expressed in the particular cell line used, the RNAi library used may lack siRNAs for that gene, the relevant siRNA may induce cytotoxicity, or by functional redundancy, other gene products in these cells might abrogate the requirement that the target gene be expressed. We introduce latent, binary *accessibility* variables 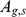 to accommodate this general effect, where 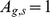 means that gene 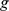 was accessible in study 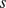, and hence, if involved and fully knocked down, would show a phenotypic effect. In the absence of more specific knowledge we treat the 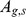
's as independent Bernoulli-distributed variables. Analysis supports allowing the accessibility rate 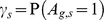 to vary among studies, and we allow this flexibility to better accommodate study-study heterogeneity.

#### Off targets

The pool of siRNAs that target gene 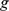 in study 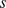 may not be fully specific, and thus may inadvertently knock down some number 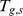 of influenza-involved off targets. These off targets are a subset of the involved off-targets associated with all siRNAs used for gene 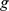 across all studies, not accounting for inaccessible genes in any given study. By modeling 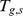 as a subset of a total, 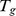, we allow potential dependencies between studies attributable to use of the same siRNA in different studies. The number 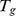 counts involved off-targets from all siRNAs for a given gene: we consider it to have mean value 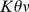, where 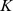 measures the (average) number of distinct siRNAs used per gene across all four studies, 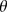 is the involvement rate, and 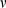 is the mean number of off-targets per siRNA. Experimental data indicates that rates of phenotypic response increase with 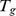, but there remain little data on the distribution of 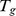 beyond computational predictions based on sequence homology [33]. From first principles, we treat 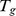 as Poisson distributed, though we investigate over-dispersed alternatives in model diagnostics. In study *s*, four (typically) siRNAs are used and these carry a subset of 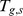 involved and accessible off-targets, having a Binomial distribution on 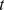 trials with success probability 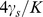 given that 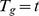. (An involved off-target that is not accessible in a given study cannot affect the phenotype in that study, even though it could do so in some other cells, for example.)

#### Dependence structure

**Figure 2:**
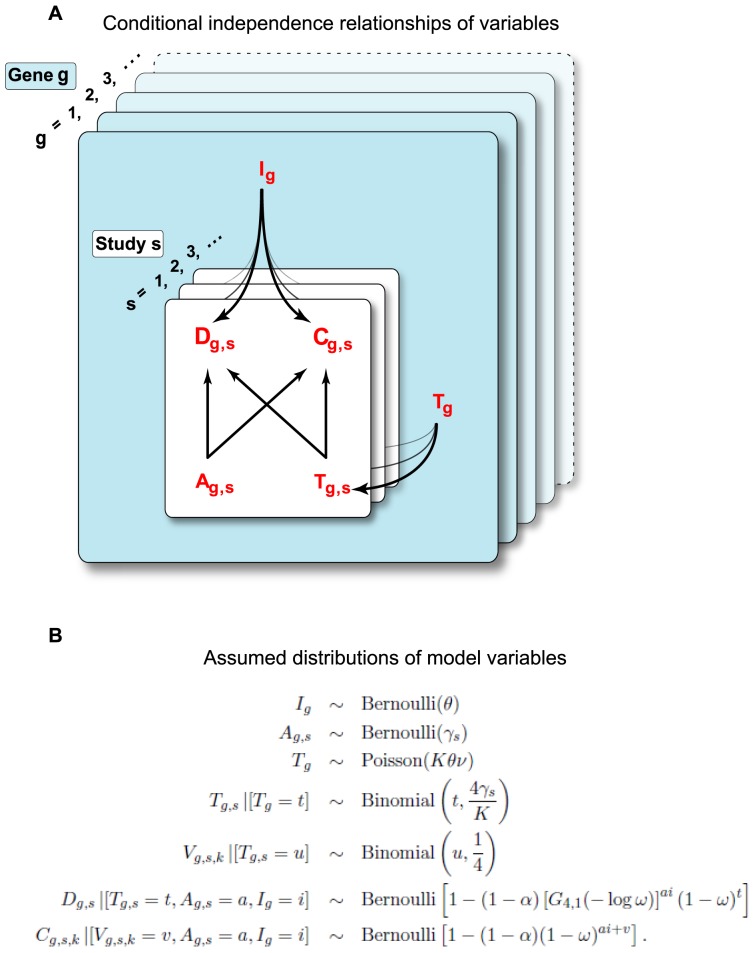
Plate diagram for statistical model. **A**) Illustrated are the conditional independence relationships of observable, reported data and latent variables in the statistical model of multiple, genome-wide RNAi studies. The white planes represent variation over studies s = 1, 2, 3, …, with each plane representing a different study. The blue planes represent variation over genes g = 1, 2, 3, …, with each plane representing a different gene. The observables for each study are 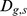 and 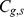, which respectively indicate whether in study s, gene g was detected in primary screening or confirmed in validation testing. The arrows indicate that the model treats 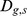 and 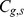 as dependent on the latent values of gene-specific variables 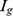 and 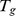, and gene- and study-specific variables 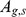 and 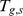. 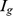 denotes whether gene g is functionally involved in influenza virus replication. 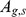 specifies the accessibility of gene g in study s, i.e., the probability that, if gene g were involved in influenza virus replication, knocking down gene g would produce a scorable phenotype. 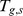 represents the number of influenza virus-involved, off-target genes that are inhibited by the siRNA(s) targeted in that study against gene g. 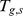 comprises a subset of 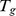, the number of influenza virus-involved off-target genes associated with all siRNAs used for gene g across all studies. **B**) Assumed distributions of the variables in the statistical model. 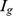 is 1 when gene g is involved in influenza virus replication, and 0 otherwise. 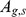 is 1 when gene g is accessible in study s. 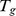 is the number of involved off-targets for gene g, relative to a pool of siRNAs that might be used to target gene g. 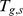 is the size of the accessible subset of 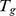. V_g,s,k_ is the size of the accessible subset of 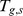 in assay k of the secondary screening in study s.

Latent factors 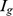, 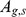 and 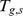 affect the distribution of observable detections 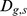 and confirmations 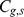. The directed acyclic graph in Figure 2 expresses the proposed model's dependence structure. For example, the probability that a gene is detected in a given study depends on whether it is truly involved in influenza virus replication, whether it is accessible in the cells used, and the number of involved and accessible off-targets (3 arrows impinging on 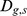.) For convenience, we assume that 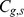 exists independently of detection, and is latent unless 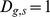. We derive results from one particular model specification, described below, and test how sensitive our conclusions are to changes in aspects of this specification.

#### Knockdown

With respect to relating phenotypic effects to siRNA action, our model is as simple as possible while allowing three basic features. First, the larger the number of either on-target or off-target events for an siRNA or siRNA pool, the higher the probability of a phenotypic effect. Second, if there are multiple off-target events from a pool of siRNAs, then distinct off-targeted genes are affected. Third, we suppose that multiple on-target hits (i.e., from multiple siRNAs targeting the same gene) deliver a higher probability of phenotypic effect than do the same number of off-target hits dispersed to various genes. A mathematical device to achieve this structure imagines that every targeting or off-targeting event (i.e. every potential knock down of an involved gene) is associated with a uniform (0,1) random variable representing the fraction of mRNA remaining after knock down by that event. An error-free measurement then would show a phenotypic effect if any of the involved, accessible genes had mRNA levels reduced below a threshold, parameterized by 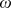 in (0,1). By assumption, off-target effects work in parallel on different genes. If 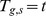, the probability that any of the off-targeted genes has mRNA knocked down below 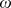 is 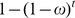. The assumptions similarly form the on-target model as a series circuit: the probability that the targeted mRNA is knocked down below 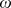 after hits from a pool of, say four, siRNAs becomes 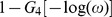, where 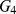 is the cumulative distribution function of a gamma distribution with shape 4 (Text S1).

#### Measurement error

Our meta-analysis analyzes summary gene-level data from four two-stage genome-wide studies. Whether or not a gene is detected or confirmed in any study depends on details of the quantitative assays used to assess the phenotypic effect, as well as on all the intrinsic factors indicated above. These assays are subject to various sources of measurement error that may create both false-negative and false-positive recordings. We allow both types, and have found improved model fits by allowing the false negative rate to be study specific. Parameters are 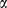 for type I (false positives) and 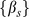 for type II (false negatives).

#### Detection model

**Figure 3:**
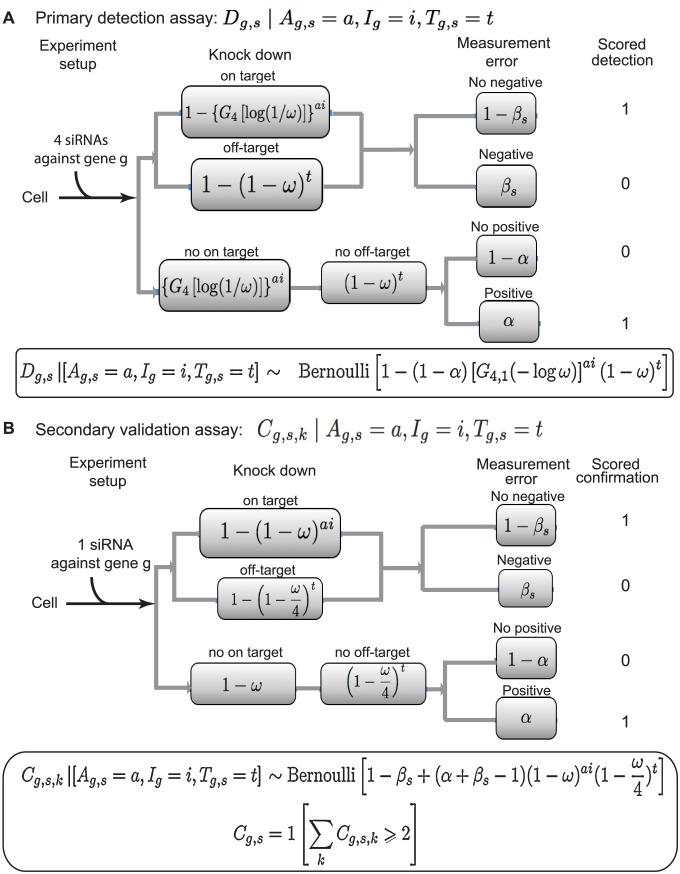
Circuit diagram representing the probability of various outcomes in two-stage RNAi screening. **A**) Detection Screen: The diagram expresses ways in which treated cells (left) can progress through the experiment to a scored detection D_g,s_, conditional upon the state of latent variables (accessibility A_g,s_, involvement I_g_, and off target count T_g,s_). In the case shown, cells are treated with a pool of 4 siRNAs against a single gene g. The top branch involves a phenotypically effective knockdown event that blocks influenza virus infection, due to an on-target effect (upper sub-branch) or off-target event (lower sub-branch) or both. The alternate bottom branch involves the absence of siRNA-mediated interference with influenza virus replication. In either case, as shown, negative measurement error (type II) or positive measurement error (type I) could affect the final scored phenotype. Depending on these combined effects, the gene g in influenza virus replication is scored as detected (1) or not detected (0). Shaded boxes record the probability of the indicated event, where variables a and i are both either 0 or 1, and count t is a natural number. Accordingly, phenotype probabilities are computed by adding probabilities over all paths from the left to the specific outcome, where a path probability is computed by multiplying over the traversed events. The open box at the bottom provides a concise summary form for the conditional detection probability. **B**) Schematic similar to (A) regarding the possible outcomes and associated probabilities for secondary confirmation testing of a given gene g implicated by detection screening in study s. Cells are treated in four separate assays with each of the four distinct siRNAs targeting gene g, and can then traverse one of the two main indicated outcome branches, similar to panel (A). As noted, confirmation requires a positive result with at least two of the individual siRNAs against gene g.

Figure 3A presents a probability model for detection 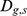 conditional upon accessibility, involvement, and off-target count. Each edge in the circuit has a probability, and the fate of cells considered prior to experimentation (left) is a path through the circuit to some end state (right). For example, a phenotypic effect (scored as 1) is possible if either (1) there is a successful knockdown of some involved gene (either on or off target) and there is no (type II) measurement error, or (2) there is neither on- nor off-target knockdown and there is a (type I) measurement error. Probabilities, which are assembled by multiplying along paths in this circuit, take a concise final form:
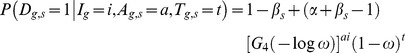
The model allows heterogeneity across genes and studies. Targeted genes that are involved 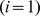 need to be accessible 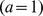, otherwise they are detected at the lower rate of non-involved genes. The constant 4 enters here because we have modeled a typical study that targets a gene by pooling four different siRNAs (with each additional siRNA improving the detection rate).

#### Confirmation model

We model secondary screen confirmations 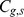 similarly to detections, but we consider a typical study in which the four individual siRNAs that had been pooled in the primary screen were applied separately in four assays. Confirmation on assay 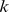 is indicated by 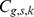, and we have 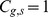 if and only if 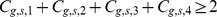; that is, if at least two of the single siRNA assays also yielded a positive phenotype (exactly as in study U2OS). Figure 3B breaks down contributions to the conditional distribution of 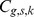; in summary, the conditional confirmation rate is:
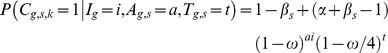



The knock-down formula is different from detections because each separate assay uses a single siRNA. See Supplementary Text S1 for further discussion. In summary, the probability distributions adopted to model the joint distribution of all the data and latent variables are recorded in Figure 2B.

#### Likelihood-based inference

The multinomial log-likelihood function is 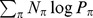, and the stated assumptions allowed us to compute pattern probabilities 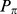 for all 81 multi-study data patterns 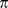 in terms of the involvement rate 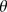, the accessibility rates 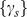, the off-target rate 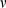, the knockdown threshold 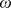, and the error rates 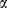 and 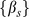. Somewhat surprisingly, the suite of latent variables (relating to involvement, accessibility, and off-targets) can be marginalized analytically (*i.e.*, their effects summed out of the likelihood function). The resulting pattern probabilities 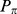 are readily computable although they are cumbersome to develop and display (see Supplementary Text S1). Numerical optimization routines (especially *nlminb* in R) enabled model fitting and the computation of maximum likelihood parameter estimates. Markov chain Monte Carlo (MCMC) was developed to sample from the posterior distribution of all parameters under a flat prior, in order to infer likely ranges for the underlying values. Results from both approaches were similar, and since the MCMC output more readily yields confidence statements, we focus on results from that computation. See Supplementary Text S2 for further details.

## Results

### Four genome wide RNAi screens identified 614 host genes important for influenza virus replication

Four nearly genome wide RNAi studies have been published to identify host genes that affect influenza virus replication (summarized in Table 1 and 2). These four studies differed in the cell lines and RNAi libraries used, but each invoked a two-stage screening strategy in which candidate genes detected in a primary, genome-wide screen (Table 1) were subjected to more thorough testing in a second, validation phase (Table 2). In such validation testing, the candidate genes were knocked down using an alternate dsRNA or multiple single siRNAs to confirm that the effects on influenza virus were not due to off-target effects on unintended genes.

**Table:** Table 1. Summary of primary screens.

Screens	DL1	U2OS	A549_DE_	A549_US_
Reference	Hao et al (2008)	Brass et al (2009)	Karlas et al (2010)	Konig et al (2010)
Cell line	Drosophila cell line	Human osteosarcoma cell line	Human lung adenocarcinoma epithelial cell line	Human lung adenocarcinoma epithelial cell line
Library	Ambion	Dharmacon siARRAY siRNA library	Qiagen Hu_genome 1.0 and Human druggable genome siRNA set V2.0	Qiagen whole genome library, Invitrogen kinome library, and IDT kinome library
siRNA	13071 *Drosophila* genes with 1 dsRNA against 1 gene	17,877 human genes with 4 siRNAs against each gene	22,843 human genes with 4 siRNAs against each druggable gene, and 2 siRNAs against each predicted gene.	19,628 human genes with 6 siRNAs against each gene
pooled siRNA	NA	Each well with 4 siRNAs	Each well with 1 siRNA	47560 wells with 2 siRNA, 3617 wells with 1 siRNA
Assay	RNAi 48 hours, infect with WSN based reporter containing FVG-R, 24 hpi, assay for luciferase	siRNA 72 hours, infect with PR8, 12 hpi, stain for HA,	siRNA 48 hours, infect with WSN, 24 hpi, stain for NP, supernatant infect 293T reporter, 16 hpi, assay for luciferase	siRNA 48 hours, infect with reporter containing WSN-Ren, 12, 24, 36 hpi, assay for luciferase
Steps in influenza virus life cycle involved	Uncoating, vRNP trafficking and nuclear import/export, genome transcription/replication, viral mRNA translation and protein trafficking	Binding, entry and fusion, uncoating, vRNP trafficking and nuclear import/export, genome transcription/replication, viral mRNA translation and protein trafficking	Binding, entry and fusion, uncoating, vRNP trafficking and nuclear import/export, genome transcription/replication, viral mRNA translation and protein trafficking, virion assembly and release	Binding, entry and fusion, uncoating, vRNP trafficking and nuclear import/export, genome transcription/replication, viral mRNA translation and protein trafficking
Repeats	2	3	3	2
Primary hits	176 *Drosophila* genes of which 143 correspond to 237 human orthologs	312	287	294
data normalization method	plate mean	plate mean	B-score* [48]	plate median
hit calling	Z score ≤−2.5	<55% of plate mean	RSA** hits with robust z-score& <−2	RSA**, p<0.4

* B-score: Like Z-scores, B scores normalize the raw data by assay variability, but also adjust the raw values for positional effects within a plate and for possible assay drift across plates within a run [48].

** RSA: Redundant siRNA activity analysis, which ranks each gene by combined analysis of the data from all siRNAs targeting that gene [49].

& Robust z-score is calculated by substituting median and median absolute deviation for mean and standard deviation in the z-score calculation [50].

**Table 2:** Summary of confirmation screens.

Screen designation	DL1	U2OS	A549_DE_	A549_US_
Primary hits tested in confirmation screening	176 *Drosophila* genes, of which 143 correspond to 237 known human orthologs	312	287	294
siRNA coverage	1 new dsRNA against each gene	4 individual siRNAs per gene	4 individual siRNAs per gene	At least 2 different siRNAs per gene
Confirmation assay	Rescreen as in the primary screen	Rescreen as in the primary screen	siRNA 48 hours, infect with WSN or Hamberg at moi 0.001, 48 hpi assay for titre	siRNA 48 hours, infect with WSN at moi 0.01, 36 hpi HA assay for titre
Confirmed by ≥2 siRNA	104 confirmed Drosophila genes, of which 96 correspondto 154 human orthologs	129	168	219
Only 1 siRNA confirmed	Not applicable	121	No data provided	59

The majority of genes detected and confirmed in these four genome-wide screens are genes that promote influenza virus replication, i.e., knock-down of these genes by RNAi decreased virus replication (Table 2). Two screens using human A549 cells identified, respectively, 219 and 168 genes that promoted influenza virus replication, without reporting any host genes that restrict influenza virus - i.e., genes whose knockdown increased viral replication. One screen in human U2OS cells found 129 genes that promoted influenza virus replication, and 4 genes that restricted replication. A screen in *Drosophila* DL1 cells identified 104 genes that promoted virus replication and 11 genes that restricted virus replication. Of the 104 *Drosophila* genes that promoted influenza virus replication, 96 have a total of 154 human homologs according to the Ensembl database, while 10 out of the 11 *Drosophila* genes that restrict influenza virus have 14 human homologs. As these studies identified very few genes that restricted virus replication when knocked down, our analysis focused on the genes that promoted virus replication. From 984 unique human genes identified in the primary screens of these four studies, 614 unique genes were confirmed that promoted influenza virus replication. The symbols and Entrez ID numbers of all 984 genes are listed in Supplemental Table S1, with unconfirmed genes at the bottom of the table in shaded rows. Thus, on average, each such study detects approximately 1% of the genes in the genome as potentially involved in the influenza virus replication, and confirms approximately half of these candidate genes.

### Studies agree substantially more at the level of functional categories than genes

Although all four studies aimed to perform a general identification of host genes affecting influenza virus replication, their gene lists exhibited relatively little overlap. Only one gene (*COPG, or coatomer protein complex, subunit gamma*) was detected and confirmed by all four studies; nine genes were confirmed by three of four studies, and 35 by two studies (Figure 1A). Pairwise overlap between studies ranged from 3% to 15%, with a mean pairwise overlap of 6.7% (Figure 1B).

**Figure 4:**
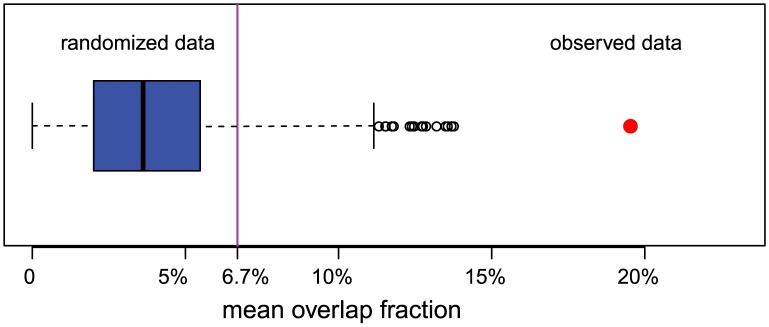
Studies agree substantially at the level of functional categories. When each study's confirmed gene list is processed for enriched functional categories from GO (Gene Ontology), substantially more categories are identified in multiple studies than one would expect by chance, suggesting a positive association in functional signals underlying the study-specific gene lists. Shown is the distribution of an agreement statistic (mean overlap fraction) generated by gene-permutation, where the multi-functional analyzer (MFA) approach is applied to every gene list to produce a list of over-represented GO terms. The actual agreement between studies (red) is strikingly higher than expected by chance (blue) and also much higher than the 6.7% gene-list agreement (purple).

To complement comparisons at the gene level, we examined the relationship of the four confirmed-gene lists at the level of functional categories recorded by the Gene Ontology (GO) project. Within-study lists of over-represented GO terms exhibited relatively strong agreement (Figure 4), with a mean overlap fraction of 19% (compared to 6.7% for gene lists). This agreement is substantially more than would be expected in the absence of functional associations between the studies (Monte Carlo p-value = 0.001). The finding is based on an advanced gene-set analysis tool which accommodates term-size and term-overlap issues (MFA; see Methods); the same conclusion was found with simpler gene-set enrichment methods of GO-term lists suggests that the four studies were probing common functional signals in influenza virus dependence on host genes.

**Figure 5:**
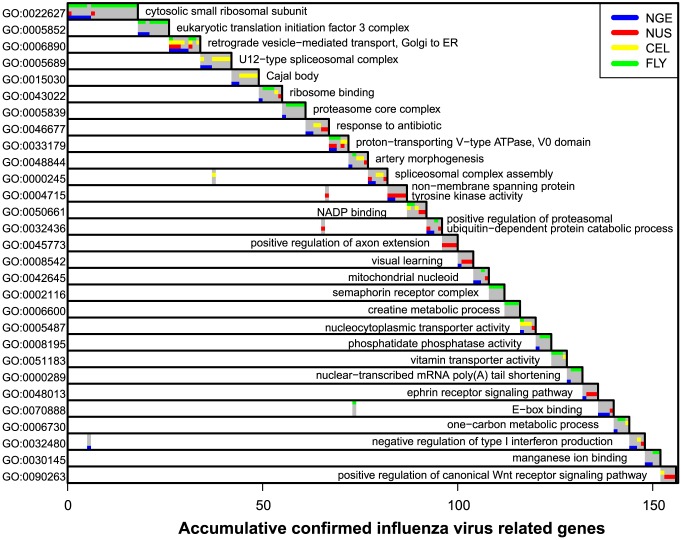
Dominant Gene Ontology terms identified in analysis of combined gene list. The multi-functional analyzer (MFA) applied to the combined list of 614 influenza-involved genes produced 91 GO terms in the maximum a posteriori (MAP) estimate of activated terms. The dominant terms are shown here, with color bars to indicate the study responsible for identifying the gene. The MFA-identified category having highest overlap with the 614-gene list is GO:0022627 (top row), the overlap being 18 genes (horizontal axis). These genes and this category are removed from the system, and then the category having the highest overlap with the remainder is identified (GO:0005852), and so on until the overlap is less than four genes. Shading in the sub-diagonal indicates genes also annotated to previously named categories.

The functional categories over-represented in the combined list of 614 confirmed genes from all four studies represent a wide variety of functions. The top 29 categories are illustrated in Figure 5, including with categories associated with mRNA translation (ribosomal small subunit, eIF3 initiation complex, ribosome binding), vesicular transport (Golgi to ER, vacuolar ATPse) RNA metabolism (RNA splicing, transport, poly(A) regulation), regulated protein degradation (proteasome, ubiquitination factors), and other functions. Cellular functions like regulation of type I interferon production and nucleo-cytoplasmic transport are known to play important roles in influenza virus replication, while other functions like NADP binding and vitamin transporter activities are novel findings identified through our analysis. Figure 5 also shows that, for many categories, two or more studies isolated distinct sets of genes in the same functional category, so that the same cellular functions were independently but repeatedly identified to promote influenza virus replication.

### Confirmed genes exhibit more physical interactions than expected by chance, both within and between studies

As noted above, if many of the genes confirmed in the genome-wide influenza screens were false positives, then we would expect these genes to be distributed across unrelated pathways and functional complexes. That is, we would expect relatively few direct interactions among the genes confirmed in one study or in different studies. Given the confirmed genes from the four influenza screens, we used known physical interactions among the genes' products, as cataloged in the BioGRID protein-protein interaction database [9], to test two specific hypotheses: First, that the genes confirmed within each individual study are more inter-related by protein interactions than would be expected by chance. Second, that there are more such relationships between genes confirmed in different studies than would be expected by chance.

To assess the relatedness of a set of confirmed genes, we first determined the number of connected components in the interaction subgraph consisting of the proteins encoded by the confirmed genes. We considered two properties of these connected components: the number of edges in the connected components and the number of genes in the largest connected component. We further measure relatedness by determining the average degree for vertices in the subgraph of confirmed genes. We also investigated augmenting our interaction data with metabolic-pathway and transcription-factor relationships, and found that the results of our analysis were qualitatively similar when we include these additional interaction sets.

To test our first hypothesis, we independently measured the above mentioned properties for the four interaction subgraphs constructed using the confirmed genes from each individual study. To determine if any of the measures was surprisingly large, we employed a Monte Carlo test with the null hypothesis that these measures could be accounted for by a randomly selected set of pseudo “confirmed” genes. Given a list of *n* actual confirmed genes, our Monte Carlo test involved repeated random selection of gene sets of size *n*. We selected these random gene sets such that the degree of connectivity of the selected genes, with respect to the entire interaction graph, was the same as the degree of connectivity of the given list of confirmed genes. The rationale for controlling the degree of connectivity in our Monte Carlo tests is that we want to rule out the possibility that a confirmed list has a high degree of connectivity with other confirmed genes simply because its genes have many known interactions.

**Figure 6:**
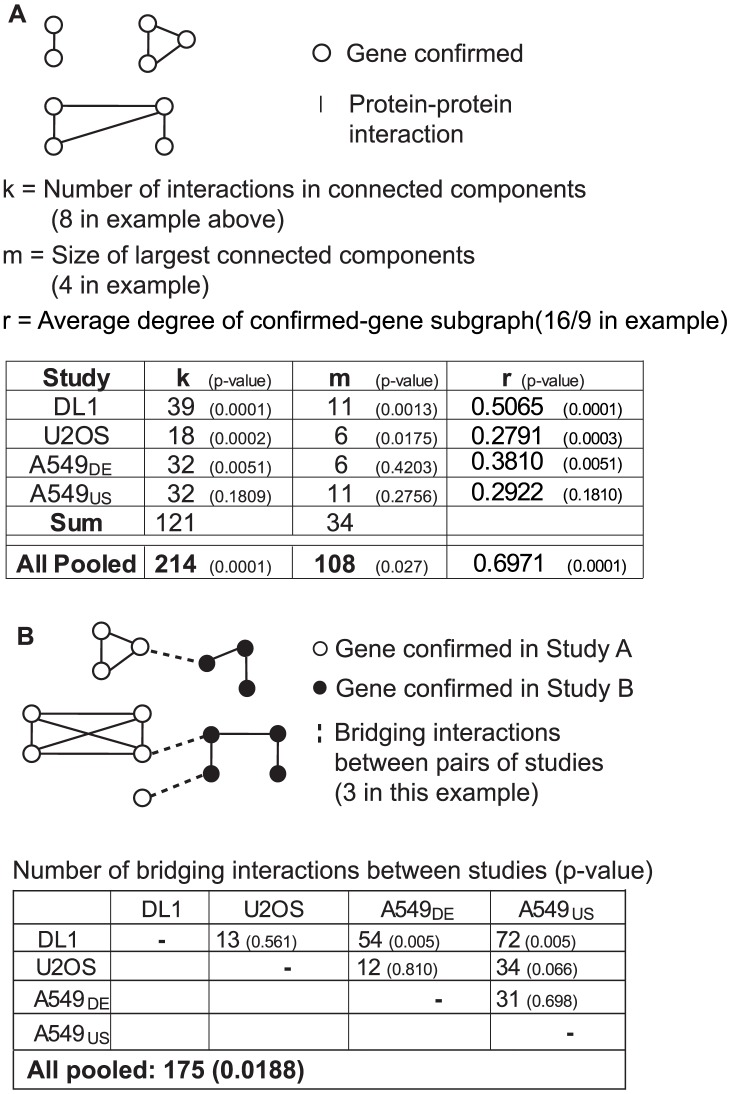
Genes implicated in different studies are related by physical interactions. **A**) Physical interactions among implicated gene products define graphs that characterize the relatedness of the genes. Each circle represents a gene confirmed in at least one RNAi study and each edge represents a protein-protein interaction. The variable k represents the number of edges in the subgraph defined by a given set of confirmed genes, m represents the number of genes in the largest connected component, and r represents the average degree of the confirmed genes in this subgraph. The values of k, m and r for each RNAi study as well as the values for the interaction subgraph resulting from pooling all four studies are shown in the table. P-values correspond to the null hypothesis that the k, m, and r values can be accounted for by randomly selected sets of pseudo “confirmed” lists. P-values were calculated based on Monte Carlo tests with 9,999 randomized data sets having the same size and interaction properties as the RNAi studies being considered. **B**) *Spanning* interactions relate genes identified in different studies. Black and white circles represent genes from two different studies, solid edges indicate protein-protein interactions relating genes identified within the same study, and dashed lines indicate spanning interactions, which link genes confirmed in different studies. The numbers of spanning interactions between pairs of studies, and among all four studies pooled, are shown in the table. P-values correspond to the null hypothesis that the counts of spanning interactions can be accounted for by randomly selected sets of “confirmed” gene lists.

Figure 6A illustrates the two connected-component measures and the average degree measure, and shows the counts and *p*-values calculated using the graphs for each individual study. For three of the four studies, the number of interactions in connected components and the average degree exceeded those of the null hypothesis by a statistically significant margin (p<0.05), and for two of the studies, the number of confirmed genes in the largest connected component is statistically significant. These results generally support our first hypothesis that the confirmed genes have a higher degree of relatedness than expected by chance.

**Figure 7:**
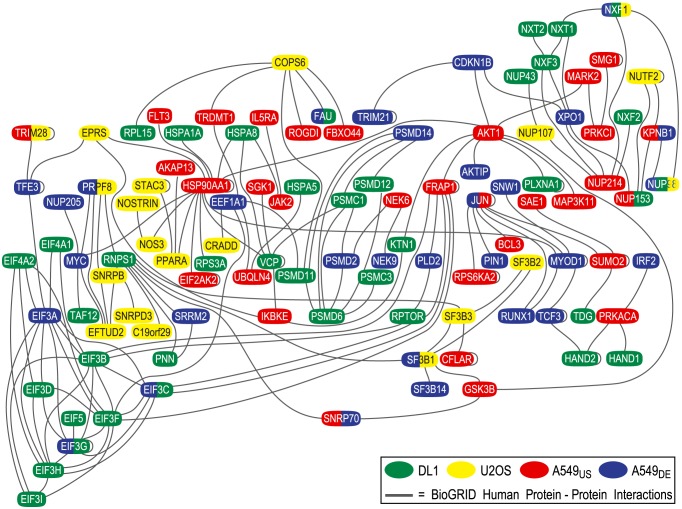
Connected component from the physical interaction network illustrates connections of four studies. The largest connected component from the physical-interaction graph which was derived by pooling genes implicated in the four RNAi studies. This connected component consists of 108 genes. Each oval represents an implicated gene, and its color (or colors) indicates the study (or studies) in which it was confirmed. Edges represent protein-protein interactions among gene products. Genes confirmed by each individual study tend not to be topologically clustered, but instead generally have interactions with genes confirmed by other studies.

To test our second hypothesis, we pooled the confirmed genes from the four independent studies and determined the connected components and the average degree in the interaction subgraph for this set of pooled genes. Figure 6A (bottom row) shows the resulting number of edges in the connected components, the size of the largest connected component, and the average degree for vertices in the pooled subgraph. The number of protein interactions in the pooled study subgraph (214) is much larger than the sum of interactions in the individual-study graphs (121), and was highly statistically significant at p<0.0001. The average degree of proteins in the pooled subgraph is larger than the average degree of any of the single-study subgraphs, and this degree was highly statistically significant (p<0.0001). Thus, these results strongly support our second hypothesis that there are more interactions between pairs of genes confirmed in different studies than expected by chance. Likewise, the size of the largest connected component in the pooled graph (108 genes) is much larger than the sum of the largest connected components in the individual-study graphs (34 genes) and was statistically significant at p<0.05. Figure 7 illustrates the 108-gene largest connected component from the pooled-study graph. As this figure indicates, the genes confirmed by each individual study tend not to be topologically clustered, but instead generally have interactions with genes confirmed by other studies.

To further probe our second hypothesis, we counted the number of spanning edges in the connected components of the pooled-study graph. A spanning edge is one that represents an interaction between two genes that were confirmed in different studies (Figure 6B). As before, we measured the statistical significance of the observed number of spanning edges by iterative Monte Carlo tests that involved randomly selecting, for each study in the pool, a set of pseudo “confirmed” genes with the same degree of connectivity as the actual confirmed genes in that study. The results show that, of the 214 total protein-protein interactions of the pooled study subgraph (Figure 6A), 175 (82%) were spanning interactions between gene products confirmed in different studies (Figure 6B). This result was statistically significant at p<0.05.

We also considered the possibility that our measures of interactivity might be inflated by the fact that some of the genes confirmed in the DL1 study mapped to multiple human orthologs. To control for this effect, we repeated all of our analyses using a smaller set of DL1 genes in which each of the 96 confirmed *Drosophila* genes was mapped to exactly one human gene. In particular, we mapped each *Drosophila* gene to the human ortholog with the *fewest* known interactors. The differences in p-values between the original analysis and this “conservative ortholog mapping” re-analysis were all small. Specifically, none of the p-values changed from statistically significant (p<0.05) to not significant.

Overall, then, the results show that the independent RNAi studies identified distinct but physically interacting sets of genes, and that these confirmed gene products exhibit significantly more interactions both within and between studies than expected by chance.

### Statistical modeling of genome-wide RNAi studies

Multiple independent analyses above show that the outputs of the four separate screens are not randomly divergent, but are highly interlinked both physically and functionally. To more thoroughly examine the biological and experimental factors that affect agreement among such screens, we generated a statistical model of RNAi screening that incorporates known biological and experimental features to better analyze and understand the expected relationships between the outputs of independent screens. The assessment of agreements and disagreements among studies has long been a focus of model-based statistical analysis, from seminal work by R.A. Fisher and colleagues on species abundance estimation in ecology [34], through more recent and relevant precursors [35]–[37] to our own calculations. The rationale for this general approach is that the specific findings of any study are affected by numerous factors, some of which are systematic and shared in some predictable way among studies, and some of which are idiosyncratic.

To capture the systematic effects we treat them as parameters in a stochastic process presumed to have generated the observed data, and we infer the parameter values by calculating the probability of observed data (the likelihood). The structure of RNAi experiments forces us to go beyond previously described probability models and propose a specification for multi-study, two-stage (detection/confirmation), genome-wide RNAi data. As described more fully in the Methods and Text S1, the model considers that a fraction 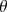 in (0, 1) of all G genes is involved in the phenotype in the sense that error-free measurements in some cell type would detect and confirm this involvement. However, both primary detections and secondary confirmations are subject to additional sources of variation. On average over the full set of siRNAs, there is a knockdown threshold parameter 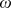 in the range (0, 1) indicating the frequency with which a single siRNA would reduce expression and function of a targeted gene sufficiently to cause detection or confirmation if the gene were involved and if knockdown and phenotypic readout were free of errors and other interfering effects (see next paragraph). Since siRNA pools show greater phenotypic penetration than an individual siRNA [38], the model further specifies how detection/confirmation rates increase as we apply more siRNAs targeting the same gene (see Methods). Figure 2A illustrates the conditional independence relationships of observable, reported data and latent variables in this statistical model, while Figure 2B shows the nature of the distributions that were assumed for each variable.

As a separate issue from the frequency of phenotypically significant knockdown at the mRNA level, we allow that many factors could block a study from detecting a phenotype associated with an involved gene. We lump these factors into study-specific accessibility parameters 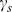
_,_ which accommodate many factors specific to cell types and conditions used in different studies. Examples of such potentially confounding factors include whether the range of genes targeted by the siRNA library used; whether the cells and conditions used for a targeted mRNA is not expressed; if the mRNA is too abundant to be cleared within the period of RNAi treatment; if the functional protein product is too long-lived to be depleted over the measurement time course; if knockdown induces general cytotoxicity, which is usually taken to disqualify scoring for specific involvement; or if the phenotypic effect of knocking down an involved gene is masked by functional redundancies such as expressed homologs, parallel pathways, etc.. All other false negative measurement errors are monitored by type II error parameters 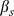, which were also made study specific since this improved model fit. Finally, the model allows false-positive detections or confirmations to occur either because of off-target effects (the parameter 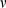 is the mean number of off-targets per siRNA) or by type I measurement error (parameter 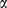). Numerical and Monte Carlo methods were used to obtain parameter estimates and approximate confidence intervals using only a set of modeling assumptions and the multi-study detection/confirmation records (Methods and Supplementary Text S1).

### Model validation by multiple diagnostic checks

We computed three simulation diagnostics to check the internal validity of the model-based inferences, as detailed in Supplementary Text S2, Section 2. First we performed a consistency check to assure the integrity of the modeling calculations and their implementation in software (Text S2-2.1). Likelihood theory predicts that maximum likelihood parameter estimates computed from ever increasing data sets converge to the underlying parameter settings when the model is identifiable. Thus, we simulated gene-level data from the model, under various parameter settings, and demonstrated that as the genome size increases these settings were recovered.

Second, we performed predictive checks to examine properties of synthetic data generated from the fitted probability model (Text S2-2.2). Table S2 in Text S2 demonstrated that simulations of the estimated generative model recapitulate statistical patterns seen in the observed data. For example, Figure S2 in Text S2 plots the number of confirmations against the number of detections, both in the observed data and in hypothetical repeats. The goodness-of-fit is compelling in this plot and in several other summaries (Table S2 in Text S2; Figure S3 in Text S2).

For the third check, we did leave-one-study-out diagnostics. We fitted the model four times, once to each subset of three studies obtained by leaving out a single study's results (Supplementary Text S2-2.3). The cross-validation findings indicate stability of the parameter estimates (Table S3 in Text S2) as well as accuracy in predictions of the left-out studies (Table S4 in Text S2).

We plotted and compared the distribution of gene counts across the 81 possible patterns (Table 3) between simulated and experimental data, and the simulation results generated from model fits accurately reflected the experimental data (Figure S7 in Text S2). Therefore, our model faithfully simulates actual RNAi studies.

**Table 3:** Multi-study data in count format.

DL1	U2OS	A549[DE]	A549[US]	*π*	*N*[*π*]
1	1	1	1	1111	21016
1	1	1	2	1112	71
1	1	1	3	1113	179
1	1	2	1	1121	106
1	1	2	2	1122	0
1	1	2	3	1123	6
1	1	3	1	1131	126
1	1	3	2	1132	2
1	1	3	3	1133	18
1	2	1	1	1211	113
1	2	1	2	1212	0
1	2	1	3	1213	1
1	2	2	1	1221	0
1	2	2	2	1222	0
1	2	2	3	1223	0
1	2	3	1	1231	2
1	2	3	2	1232	0
1	2	3	3	1233	0
1	3	1	1	1311	111
1	3	1	2	1312	1
1	3	1	3	1313	3
1	3	2	1	1321	2
1	3	2	2	1322	0
1	3	2	3	1323	0
1	3	3	1	1331	3
1	3	3	2	1332	0
1	3	3	3	1333	3
2	1	1	1	2111	80
2	1	1	2	2112	0
2	1	1	3	2113	2
2	1	2	1	2121	0
2	1	2	2	2122	0
2	1	2	3	2123	0
2	1	3	1	2131	1
2	1	3	2	2132	0
2	1	3	3	2133	0
2	2	1	1	2211	0
2	2	1	2	2212	0
2	2	1	3	2213	0
2	2	2	1	2221	0
2	2	2	2	2222	0
2	2	2	3	2223	0
2	2	3	1	2231	0
2	2	3	2	2232	0
2	2	3	3	2233	0
2	3	1	1	2311	0
2	3	1	2	2312	0
2	3	1	3	2313	0
2	3	2	1	2321	0
2	3	2	2	2322	0
2	3	2	3	2323	0
2	3	3	1	2331	0
2	3	3	2	2332	0
2	3	3	3	2333	0
3	1	1	1	3111	127
3	1	1	2	3112	1
3	1	1	3	3113	2
3	1	2	1	3121	4
3	1	2	2	3122	0
3	1	2	3	3123	0
3	1	3	1	3131	6
3	1	3	2	3132	0
3	1	3	3	3133	3
3	2	1	1	3211	4
3	2	1	2	3212	0
3	2	1	3	3213	0
3	2	2	1	3221	0
3	2	2	2	3222	0
3	2	2	3	3223	0
3	2	3	1	3231	1
3	2	3	2	3232	0
3	2	3	3	3233	0
3	3	1	1	3311	2
3	3	1	2	3312	0
3	3	1	3	3313	0
3	3	2	1	3321	0
3	3	2	2	3322	0
3	3	2	3	3323	1
3	3	3	1	3331	2
3	3	3	2	3332	0
3	3	3	3	3333	1

The right-most column shows the number of genes 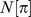 having the named pattern 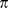 of detections and confirmations. For each study, (1) means not detected in the primary screen, (2) means detected in the primary screen but not confirmed in the secondary screen, and (3) means detected in the primary and also confirmed in the secondary screen. The first row counts genes that were never detected, and assumes a full genome size G = 22000.

### Estimation of parameters governing the output of RNAi studies

Table 4 reports all parameter estimates in the fitted model. The threshold parameter 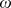 for the frequency of effective knockdown at the mRNA level was estimated to be 75–99% (95% CI), which indicates that there is a fairly high chance for an involved gene to be scored as positive, if the gene is accessible and measured in an error free system. The largest factor affecting inter-study agreement was the general accessibility rate 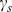, which we estimated separately for each study to improve model fit, and which ranged from 5–17% (95% CI across all studies). In other words, these results imply that, in a typical genome-wide RNAi study, most influenza virus-involved genes are inaccessible because, as noted above, one or more of many potential confounding factors either blocks knockdown or interrupts the transfer of this effect into a measurable phenotype. Biological factors and measurements consistent with such limited genetic accessibility are considered in the Discussion.

**Table 4:** Parameter estimates.

Parameter	MLE	Posterior Mean	95% CI
θ	0.128	0.126	(0.101,0.158)
α	0.003	0.003	(0.002,0.004)
β DL1	0.112	0.159	(0.011,0.338)
β U2OS	0.360	0.398	(0.267,0.502)
β A549_DE_	0.333	0.372	(0.246,0.470)
β A549_US_	0.067	0.122	(0.010,0.264)
γ DL1	0.065	0.072	(0.050,0.103)
γ U2OS	0.097	0.107	(0.075,0.145)
γ A549_DE_	0.116	0.127	(0.091,0.169)
γ A549_US_	0.086	0.095	(0.069,0.126)
ω	0.834	0.900	(0.754,0.996)
ν	0.000	0.009	(0.000,0.032)
Derived			
N	2821	2766	(2306,3342)
FDR	0.000	0.005	(0.000,0.017)
FNDR	0.122	0.120	(0.095,0.152)
FP	0.000	0.000	(0.000,0.001)
FN	0.945	0.944	(0.927,0.958)

In model of multi-study gene-level influenza data: θ is the overall rate of genes involved with influenza; α represents false positive measurement errors, and β measures false negative errors, allowing heterogeneity among studies; γ measures the rate at which genes are accessible for knockdown; ω measures how well mRNA knock down is achieved in error free recordings, and ν measures the average number of off-targets per siRNA. Estimates are based on maximum likelihood (MLE) as well as by Bayesian posterior averaging under a flat prior. Estimates of derived quantities are shown in the lower rows.

Another study-specific error causing false negatives is type II measurement error, which depending on individual study setup contributes 1–50% false negatives (95% CI across all studies). By contrast, both parameters dictating the frequency of false positives were estimated to be low. The type I measurement error 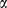 is estimated at 0.3%, which corresponds to approximately one false-positive well per 384-well plate. The off-target rate 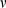 is further discussed in the next section.

### Off-target effects and experimental confirmation rates

Besides type I measurement error, false positives can also result from knocking down an unintended gene during RNAi, i.e., the off-target effect. In our model estimation, the off-target rate 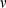 was lower than initially expected, with an estimated average of up to 0.032 off-target genes per siRNA. We wondered if this finding might be attributable to our use of a Poisson distribution model for the number of off-targets per siRNA and performed robustness checks to test this assumption (Supplementary Text S2-2.4). Little is known about the details of off-target distributions, although computational predictions from a Drosophila dsRNA library suggest a highly non-Poisson structure [33]. In fact, the Poisson assumption made little difference for parameter estimation, as evidenced by computations done under a highly over-dispersed negative-binomial alternative to the Poisson (Supplementary Text S2-2.4).

**Figure 8:**
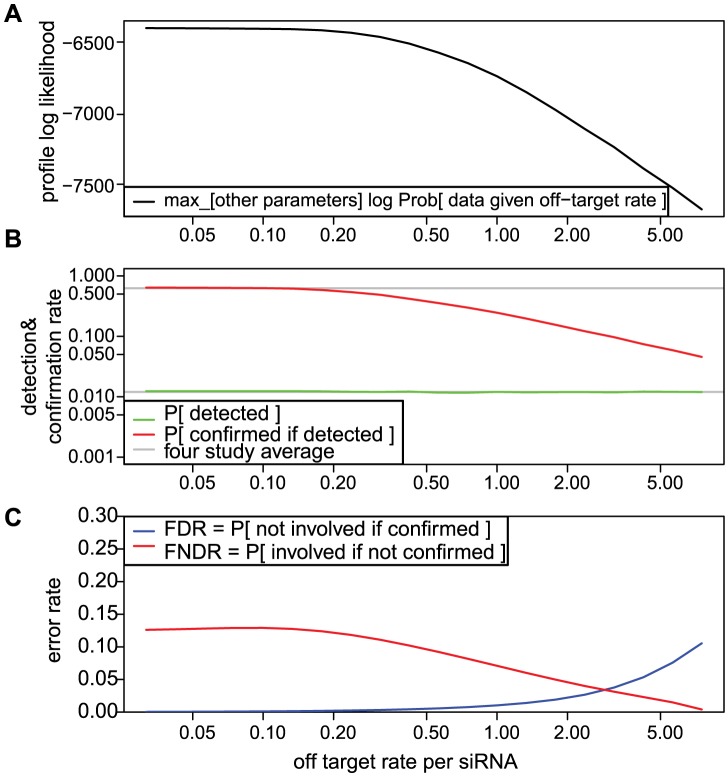
Likelihood estimation prefers a small off-target rate. Maximum likelihood estimates (MLEs) are parameter settings that provide the best explanation for observed multi-study agreement counts, yet the estimate for ν, the mean number of off-targets per siRNA, is surprisingly small (Table 4). Profile likelihood provides additional insight into this effect. At each fixed ν on a fine grid we found MLEs of the remaining system parameters as well as the maximum (log) likelihood (Panel A). The decreasing profile likelihood re-expresses the inference that fit decays if we insist on moderate to large values for ν. One reason for the poor fit is seen in a comparison of (study averaged) detection and confirmation rates computed at the profile MLEs (Panel B). Although the best fitting detection rate (green) matches the empirical detection rate regardless of ν, the best fitting confirmation rate (red) is much lower than its empirical counterpart. The profile MLE values can also be converted to false discovery rates (FDR) and false non-discovery rates (FNDR), as shown in Panel C. In the regime preferred by the observed multi-study counts (small ν), false negatives dominate, but the opposite inference follows if ν is large.

The multi-study data prefer a small off-target rate, as shown by the profile likelihood plot for this parameter in Figure 8A. In this calculation, we fixed 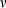 at various sub-optimal values and computed maximum likelihood estimates of the remaining parameters using the multi-study RNAi data. A source for the lack of fit is shown in Figure 8B: increasing 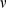 above its maximum likelihood estimate the model cannot explain the relatively high observed confirmation rate among detected candidate genes (mean rate over 50%). Figure 8C shows the effect on key error rates in fixing 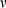 at sub-optimal values.

### False negatives are more prevalent than false positives

**Figure 9:**
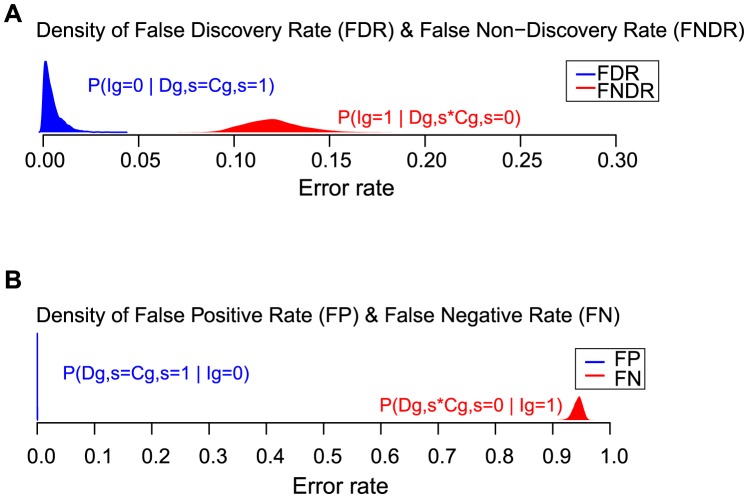
False negative rate exceeds false positive rate. **A**) Posterior distributions of false discovery rate (FDR) and false non-discovery rate (FNDR) estimated by Markov chain Monte Carlo using multi-study influenza RNAi data and the statistical model of multi-study agreement. The false positive rate FDR is the probability that a gene is not truly involved (Ig = 0) if it is confirmed in one study, not accounting for what may happen in other studies. Similarly the false negative rate FNDR is the probability that a gene is truly involved (Ig = 1) if it is not confirmed Evidently the false negative rate is higher than false positive rate according to these posterior distributions. **B**) Similar to Panel A, though false positive (FP) and false negative (FN) rates are defined relative to the true gene involvement status rather than to lists of confirmed genes.

False-positive and false-negative rates can be defined in various ways, depending on the reference set of genes. Using estimates and uncertainties in the model parameters, we estimated the false discovery rate (FDR), the false non-discovery rate (FNDR), the false positive rate (FP) and the false negative rate (FN), and we obtained posterior distributions for each in order to get approximate confidence intervals (Figure 9). FDR, which is widely used in high-throughput studies, is the rate of false positives among all statistically significant findings. In the case of the influenza study, the FDR is the probability that a gene is not involved with influenza given that this gene was confirmed in a secondary screen to be involved (i.e., the denominator counts the number of confirmations). Similarly FNDR is the probability of involvement given the gene is not confirmed (whether or not it is detected). For design purposes it is often useful to think of errors relative to the true set of involved genes (FN) or non-involved genes (FP). By either sets of measures, the clear indication from Figure 9 is that false-negative factors dominate false-positive factors in explaining the limited agreement among studies. Thus, the low overlap among the confirmed gene lists of the different studies arises principally due to missed genes.

### Estimated total number of virus-involved cellular genes

**Figure 10:**
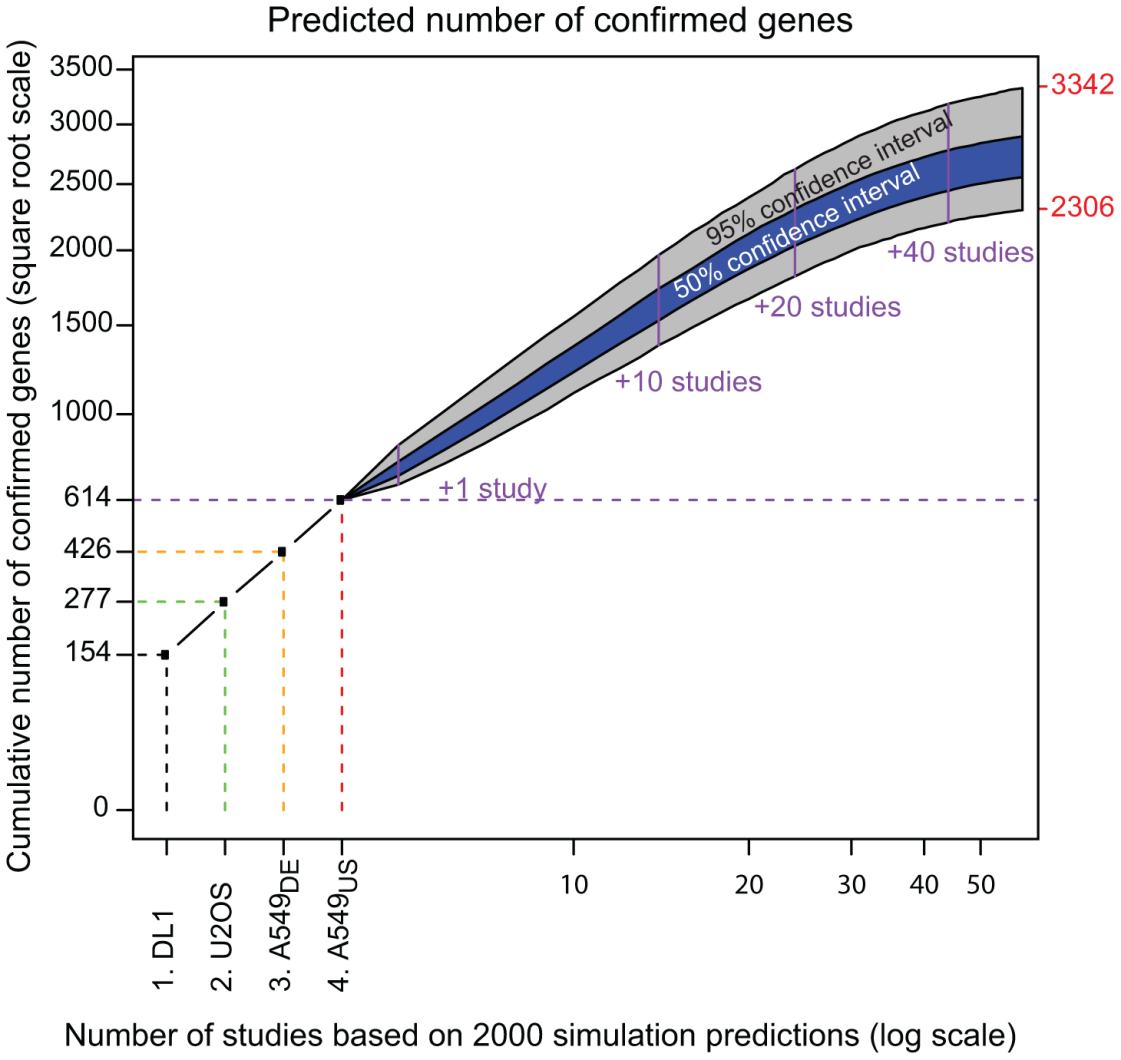
Prediction of findings in similar future studies. If the four analyzed RNAi studies are the first in a possibly long sequence of similar genome wide efforts, one could ask what is likely to happen in the remaining studies. An answer is possible via posterior predictive inference, which accounts for both random fluctuations inherent in study-specific data as well as uncertainty in the system parameters. Summarized above are predictions from 2000 simulated study sequences, with each sequence determined by a parameter setting obtained by Markov chain Monte Carlo and subsequently with future-study counts simulated prospectively from the specified multinomial model. The number of confirmed genes necessarily increases but stabilizes after 12 studies to a range consistent with the inferred number of influenza-involved genes (indicated in red number, as CI 95%). Grey and blue bands express different levels of confidence.

Since the above results infer that the low gene level overlap between and among the four documented studies (Figure 1) is primarily due to false negatives (Figure 9), the total number of involved genes is expected to be significantly more than the 614 confirmed in these initial studies. Our model estimated the rate of gene involvement in influenza virus replication (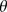) at 12% of the genome. Taking G = 22,000 total genes, this corresponds to N = 2766 genes, with a 95% posterior confidence interval of (2306, 3342). Supplementary Text S2-4 and Figure S8 in Text S2 provide further details on this inference. Figure 10 shows the predicted progression in additional confirmations beyond the 614 initial genes if further RNAi studies of similar in design were performed (details in Supplementary Text S2-4). As illustrated in Figure 10, a total of ∼12 studies would be required to identify the majority of host genes involved in influenza virus replication.

## Discussion

### Individual genome-wide RNAi studies each identifies a subset of relevant genes

Although individual genome-wide RNAi studies typically implicate from one to several hundred genes in a given biological process, multiple independent findings in our results indicate that such studies typically miss most involved genes due to a high false-negative rate during primary genome-wide screening. Thus, the results of each study provide a partial glimpse into a larger, interconnected whole. For the influenza virus studies examined here, e.g., although the gene level agreement between RNAi studies is relatively low (mean overlap fraction 6.7%), much higher levels of agreement between studies (19%) exist at the level of functional categories (Figure 4). Further, we found that the gene sets implicated in independent studies are highly connected by interactions among their protein products (Figures 6–7). These analyses examined the implicated genes from different perspectives, but all indicated that the gene sets identified in independent studies are significantly related. We conclude that, in general, functional pathways are better represented than individual genes in genome-wide RNAi studies, that independent RNAi studies add value, and that single RNAi studies can be valuable, particularly if interpreted in light of these and other insights.

### Modeling confirms that false negative errors exceed false positives

Using the measurements from the four influenza virus studies, we developed a statistical model to estimate critical parameters that control the output of genome-wide RNAi studies. The model was designed to reflect the experimental process of RNAi screening and to utilize the available published information from such screens. Our model was refined through several rounds of iteration and fits the experimental data very well (Table S2 in Text S2, Figure S2, S3 and S7 in Text S2). The leave-one-out test showed that the range and medians of the model estimations match the real experimental data well (Table S3 in Text S2).

In addition to other insights, our statistical modeling confirmed that an individual genome-wide RNAi study generally will only identify a small portion of genes implicated in a biological process. The model further indicates that this partial coverage results predominantly from false-negative errors in the high-throughput, genome-wide primary gene detection phase of the analysis, and that false positives in the more focused, repeated validation phase of analysis are much rarer. Below we discuss factors related to these false-positive and false-negative rates.

As noted above, many issues might lead to false discoveries in a genome-wide high-throughput screen. However, to reduce false discoveries, all four studies in our meta-analysis incorporated validation testing of implicated genes with independent siRNAs and multiple repeats. Stringent statistics were used to control the false discovery rate, normally to below 5%. Accordingly, such validated genes, although only confirmed in one study, are unlikely to be false positives.

A potentially significant issue causing false positives for any RNAi knockdown study (either single gene or genome-wide) is the possibility of off-target effects [15], [16], [33], [39], [40]. In most genome-wide RNAi studies, including the four analyzed here, the potential for off-target effects in the initial screening phase is addressed by requiring the knock-down phenotype to be independently confirmed by independent, usually multiple tests with two or more distinct siRNAs against the target gene. As siRNAs with distinct sequences are unlikely to affect overlapping off-target genes, such testing considerably reduces the potential for off-target induced false positives. Indeed, the estimated off target rate was low, according to model based calculations (Table 4). Our further analysis showed that if we forced the off-target rate to be higher, the model fit the data poorly and did not explain the observed confirmation rates of >50% of the detected genes. Increasing the off target rate to ∼5 genes/siRNA, e.g., shifts the estimated confirmation rate to 5% or less, which contradicts the data from all four studies (Figure 8B), and amplifies the estimated gene involvement rate to over 80%, which appears unlikely from many practical and biological considerations. The actual model-estimated rate of host gene involvement of 12% (Table 4, 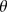), while higher than predicted from individual studies, is consistent with the many empirical and *a priori* considerations supporting the conclusion that true negatives should significantly outnumber true positives in such screens for viral dependency factors.

By comparison, the sheer scale and technical challenges of genome-wide studies presently make false negatives hard to control in the initial detection phase of global RNAi studies. Because of the large number of samples to test (typically tens of thousands), only limited replicates (typically 2 or 3) can be performed in the primary, genome-wide screens. Furthermore, simultaneous processing of hundreds to thousands of samples in microwell plates makes it impossible to individually optimize many critical parameters, such as assay timing, for knockdown analysis of each gene. Moreover, choices made to facilitate such high-throughput analysis, such as tractable endpoints, assays or virus genotypes may further affect the ability to detect at least some phenotypic effects. These and other factors noted below, including genetic redundancies, can restrict detection sensitivities and reduce phenotypic impacts, jeopardizing the detection of potentially positive phenotypes against the background of often significant experimental noise in such large-scale, high-throughput measurements.

Indeed, relative to most cell-based screens, screens for effects on viral replication are subject to additional variability associated with fluctuations in the efficiencies of initial and later stages of infection. Notably, substantial portions of such variations in infection efficiency are correlated with cell density, cell size and other local population features of the cultured cells, and appropriate normalization for such factors can significantly improve the consistency of the assay results [24], [25]. In addition to such within-screen variability, and as noted in part above, variability between independent screens is increased by differences in viral genotypes, cells, culture conditions, assay design (partial or full virus lifecycle, direct or indirect readout, timing, etc.), and similar experimental issues (Table 1). This combination of issues, plus additional sources of variable and systematic errors noted in the Introduction, makes the use of appropriate quality control practices and analytical methods [27] particularly critical for virus-oriented screens.

### Low gene accessibility is a major obstacle for identifying implicated genes

Besides the above technical challenges, our statistical inference indicates that a major contributing issue to false negatives is low gene accessibility, i.e., the portion of genes for which the method of a given RNAi study can show a corresponding loss-of-function phenotype if the gene is involved. Specifically, our fitted statistical model estimated from the data of the four influenza virus studies that only ∼10% of tested genes could be effectively assayed by RNAi knockdown for phenotypes in any one study (Table 4).

This statistically inferred low gene accessibility is consistent with and presumably results from multiple aspects of host cell biology. First, genes not expressed in the specific cell type used for an RNAi study will not show any phenotype upon attempted knocked down. In analyses of gene expression in 84 human tissues and cell lines using high coverage Affymetrix DNA microarrays, only 37% of total probes were found to have moderate or higher expression in at least one cell type [41]. These probes mapped to 9214 different genes, and the number of genes expressed in each cell type ranged from 484 (ovary tissue) to 6038 (B lymphoblast). In lung tissue, relevant to influenza virus infection, only 3355 genes, or ∼15% of the total were expressed [41]. Thus, in many cell types, a relatively small fraction of genes may exhibit sufficiently high expression to produce measurable phenotypic effects. Moreover, the particular sets of genes that are functionally expressed vary between different tissue and cell types in vivo, and between different cells and cell lines in culture [41]. Accordingly, RNAi screening in different cell lines will reveal contributions from different genes, either directly because of differences in the expression of particular tested genes or indirectly, because the contribution of a tested gene can be masked by other complementing genes that are differentially expressed between the cell lines (see also below).

Second, in genome-wide RNAi studies, genes that cause cytoxicity when knocked down are generally excluded from analysis. In the A549_DE_ study [8], e.g., 1520 siRNAs were judged to be cytotoxic and were omitted from further analysis.

Third, as cells have been selected for functional robustness, knocking down expression of a single gene may have little phenotypic effect when additional genes provide similar functions. Multiple mechanisms for such genetic redundancy have been recognized [42], [43], including homologous genes with overlapping functions, parallel metabolic or regulatory pathways, and other processes. Such buffering effects appear to be common since many genes are members of multi-gene families and many examples are known where two or more genes must be simultaneously inhibited to produce a phenotype.

Finally, the phenotypic efficiency of an siRNA depends on many factors including the level of the targeted mRNA [44] and the half-life of its encoded protein. Notably, many proteins with half-lives in days cannot be sufficiently depleted within the time frame of an RNAi screen [39]. All of the above factors combine to mask phenotypes and reduce the number of genes that can be characterized through current RNAi screening.

### Implications for further genome-wide RNAi studies

Our results provide useful approaches for conducting and interpreting genome-wide RNAi studies. Our statistical model provides a framework to describe a complicated biological assay, genome-wide RNAi, by modeling factors that can contribute to the final output. Such factors include general measurement errors that affect many assays, as well as factors specific to RNAi, such as off-target effects and gene accessibility. The model structure is not specific to influenza virus or even to virus infection. Thus we expect that the approach is applicable to genome wide RNAi studies of many cellular phenotypes. To test this, we applied the model-based estimation method to a collection of three genome-wide RNAi studies for HIV host factors (Supplementary Text S2, Section 5). The resulting HIV-derived parameter estimates were comparable to those from influenza virus, although an even lower overlap among the implicated gene sets led to a higher estimate of the involvement rate 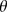, at 28% (Supplementary Text S2, Table S6 in Text S2).

As noted above, multiple findings imply that a key contributing factor to false-negative results is low gene accessibility. In future studies this could be addressed by performing screens in a variety of cell lines and possibly by expanding the time points and assay conditions used. In addition, insightful bioinformatics analysis with implicated genes could expand interpretation of host factors involved in virus infection by integrating information from other genome-wide or single gene studies. Insights from such analysis could be used to focus future studies on selected pathways to further test their involvement. Useful examples of such bioinformatic extensions include recent studies analyzing host genes required for HIV replication in the context of protein-protein interaction and other networking properties [45]–[47]. These studies offer experimentally useful insights into the functional organization of such genes and their interactions with HIV and, like the results presented here, also explicitly suggest that many host factors required for HIV replication remain to be discovered.

Additional information will help to refine the working probability model and thus provide a more accurate description of genome-wide RNAi studies. Our model was necessarily based on available published data, which was generally limited to lists of genes implicated in the primary detection screen and confirmed in the secondary validation test. To enrich understanding and modeling such RNAi screens, we strongly support publishing full genome-wide data from such studies, including data on genes excluded either for cell toxicity or low expression.

## Supporting Information

Table S1 **Implicated genes identified from the 4 studies used in the meta-analysis.** Genes identified from the 4 RNAi studies are listed in this table. The whole list contains genes identified from primary screen for secondary confirmation with the confirmed genes on top and the un-confirmed genes below in shade.
(XLSX)

Text S1 **Statistical model development and computational approach.**
(PDF)

Text S2 **Statistical model validation, inference, and prediction.**
(PDF)
