# Supplementary material for: Limited Agreement of Independent RNAi Screens for Virus-Required Host Genes Owes More to False-Negative than False-Positive Factors
Source: PLoS Comput Biol. 2013 Sep 19;9(9):e1003235. doi: 10.1371/journal.pcbi.1003235 (PMC3777922; doi:10.1371/journal.pcbi.1003235)
Supplement: Text S1 — Statistical model development and computational approach. (PDF) [file pcbi.1003235.s002.pdf]

Supplementary Material

**Text S1:**	Statistical Model Development and Computational Approach
**Main paper by:**	Hao, L, He, Q, Craven, M, Newton, MA, and Ahlquist, P
**Date:**	May 21, 2013
**Contact:**	Michael A. Newton, `newton@stat.wisc.edu`

1 Outline

Regarding the statistical model introduced in the main paper, this document provides additional details on the probability of observed patterns of detection and confirmation over four studies. It also describes our computational approach to likelihood-based inference via numerical optimization and Markov chain Monte Carlo (MCMC).

The assessment of agreements and disagreements among studies has long been a focus of model-based statistical analysis, from seminal work by R.A. Fisher and colleagues on species abundance estimation in ecology (Fisher *et al.* 1943) to more recent and relevant precursors to our own calculations, including Raftery (1988), Craig *et al.* (1997), and Basu and Ebrahimi (2001). The rationale for this general approach is that the specific findings of any study are affected by numerous factors, some of which are systematic and shared in some predictable way among studies, and some of which are idiosyncratic. To capture the systematic effects we treat them as parameters in a stochastic process presumed to have generated the observed data, and we infer the parameter values by calculating the probability of observed data (the likelihood). The design and structure of RNAi experiments forces us to go beyond previously described probability models and propose a specification for multi-study, two-stage (detection/confirmation), genome-wide RNAi data.

2 Model specification and pattern probabilities

In their essential form, data consist of binary indicators

$$\begin{aligned} D_{g,s} &= 1\,[\text{gene } g \text{ is detected by the primary screen of study } s\ ]\,, \\ C_{g,s} &= 1\,[\text{gene } g \text{ is confirmed by the secondary screen of study } s\ ] \end{aligned}$$

for genes $g = 1, 2, ..., G$ and studies $s = 1, 2, 3, 4$. We consider the genome to be the union of genes that are covered by the siRNA libraries used in the four studies, and assume the genome size $G = 22000$. We observe detection indicators $\{D_{g,s}\}$ for all g and s. We observe confirmation indicators $\{C_{g,s}\}$ only for cases g and s where $D_{g,s} = 1$. That is, the primary screen of each study is viewed as scanning the full genome; the secondary screen aims to confirm those primary findings. It is technically convenient to allow $C_{g,s}$ to be defined even when $D_{g,s} = 0$, though by the study designs such $C_{g,s}$ is unobserved and does not enter our computations. The four studies differ in details of their secondary screens. To simplify our analysis we model studies similarly, in terms of results $C_{g,s,k}$ from further assays $k = 1, 2, 3, 4$, wherein the kth assay entails the application of the kth siRNA from the pool of (typically) 4 siRNAs that targets gene g. Then we have confirmation $C_{g,s} = 1$ if (and only if) at least two of these assays indicates a phenotype; i.e. if $\sum_k C_{g,s,k} \geq 2$. Several studies (e.g., U2OS) have this precise structure, although we have not used any assay-level data $C_{g,k,s}$ in subsequent computations (these data are not complete; we use the summary calls $C_{g,s}$). Not all studies have this structure (e.g., DL-1); the key consequence of modeling this way is that the secondary validation is more stringent for filtering non-involved genes.

There are three observable states of $(D_{g,s}, C_{g,s})$ for a given gene g in a given study s:

$$\{(0,0), (1,0), (1,1)\}.$$

The proposed model entails gene-specific latent random effects, and thus marginally the $(D_{g,s}, C_{g,s})$ is not independent from $(D_{g,s'}, C_{g,s'})$ for any two studies s and s'. Our model does entail independence among genes, and therefore the likelihood (probability of observed data) can be expressed as the probability of the multinomial count vector $\{N_\pi\}$ over the $3^4 = 81$ possible multi-study observation states, or *patterns*, $\{\pi\}$ (see Table 3), where

$$N_\pi = \sum_{g=1}^{G} 1\left[\{(D_{g,s}, C_{g,s})\} \text{ has pattern } \pi \ \right].$$

(On the independence among genes assumption, this is conditional on involvement (see below) and expresses the fact that separate cells and assays are used for different genes within a given study.) In these terms, the log-likelihood is

$$\mathcal{L} = \log \mathrm{Prob}(\text{data}) = \sum_{\pi} N_\pi \log P_\pi, \tag{1}$$

where pattern probabilities $\{P_\pi\}$ are defined by a smaller number of parameters through a stochastic model of genome-wide RNAi. The model is hierarchical, and is specified using latent random effects:

$$\begin{aligned}
I_g &= 1[g \text{ is } \textit{involved} \text{ in influenza virus replication}] \\
A_{g,s} &= 1[g \text{ is } \textit{accessible} \text{ in study } s\ ] \\
T_g &= \text{number of involved off-}\textit{targets} \text{ for gene } g\text{, relative to a pool of siRNAs} \\
& \text{ that might be used to target gene } g \\
T_{g,s} &= \text{size of the accessible subset of } T_g \text{ in study } s \\
V_{g,s,k} &= \text{size of the accessible subset of } T_{g,s} \text{ in assay } k \text{ of secondary screen in study } s\ .
\end{aligned}$$

One could alternatively classify the $\{I_g\}$ as a high-dimensional parameter, but in doing Bayesian inference we would immediately cover it with a prior, so we treat it as a vector of latent factors in the notation.

A number system-level parameters are used to specify the probability structure of latent variables and observed data; they describe the basic system in terms of rates governing the latent variables as well as quantities affecting false-positive and false-negative detections and confirmations:

$$\begin{aligned}
\theta &= \text{proportion of genome involved in influenza virus replication} \\
\alpha &= \text{false positive measurement error} \\
\beta_s &= \text{false negative measurement error of study } s \\
\gamma_s &= \text{rate at which genes are accessible in study } s \\
\omega &= \text{expression threshold in knockdown model} \\
\nu &= \text{average number of off-target genes per siRNA}\ .
\end{aligned}$$

The log-likelihood $\mathcal{L}$ in (1) is a function of these 12 parameters, which we collect in a vector $\psi = (\theta, \alpha, \beta, \gamma, \omega, \nu)$, where $\beta = \{\beta_s\}_{s=1}^4$, $\gamma = \{\gamma_s\}_{s=1}^4$. Thus $\mathcal{L} = \mathcal{L}(\psi)$. The stochastic model itself

is:

$$
\begin{aligned}
I_g &\sim \text{Bernoulli}(\theta) \\
A_{g,s} &\sim \text{Bernoulli}(\gamma_s) \\
T_g &\sim \text{Poisson}(K\theta\nu) \\
T_{g,s}\,|[T_g = t] &\sim \text{Binomial}\left(t, \frac{4\gamma_s}{K}\right) \\
V_{g,s,k}\,|[T_{g,s} = u] &\sim \text{Binomial}\left(u, \frac{1}{4}\right) \\
D_{g,s}\,|[I_g = i, A_{g,s} = a, T_{g,s} = t] &\sim \text{Bernoulli}\left[1 - \beta_s + (\alpha + \beta_s - 1)\left[G_{4,1}(-\log\omega)\right]^{ai}(1-\omega)^t\right] \\
C_{g,s,k}\,|[I_g = i, A_{g,s} = a, V_{g,s,k} = v] &\sim \text{Bernoulli}\left[1 - \beta_s + (\alpha + \beta_s - 1)(1-\omega)^{ai+v}\right].
\end{aligned}
\tag{2}
$$

$G_{4,1}(.)$ is the c.d.f. of a gamma distribution with shape parameter 4 and scale parameter 1. Another system-level parameter we fix a priori and do not estimate from the data is

$$K = \text{number of siRNAs that target a gene, in total over studies.}$$

We're modeling a typical gene targeted by 4 siRNA's in the detection screen of each study. If all studies happen to use the same siRNA library, then there would be only $K = 4$ siRNA's used alltogether for this gene across the studies. On the other hand, increasing K corresponds to a wider diversity of siRNA's targeting a given gene and ultimately to different siRNA's per study. K controls the correlation in numbers of off-targets between studies, i.e. $\mathrm{cor}(\mathrm{T_{g,s}}, \mathrm{T_{g,s'}}) \to 0$, as $K \to \infty$. In our case, we fix $K = 12$ since the 4 studies of interest use 3 different libraries. Diagnostic computations showed little sensitivity to this setting.

A full specification of conditional independence assumptions is in Figure 2. The various modeling elements have been introduced to address known features of genome-wide siRNA screening. For example, every additional siRNA applied to an involved gene increases the chance that the harboring cells exhibit a phenotype. The higher the rate of involved genes, the higher the rate of an off-target phenotype. There is heterogeneity among genes, owing to whether or not they are involved, and owing to varying amounts of off-targets associated with their targeting pools of siRNAs, but there is among-gene independence in terms of siRNA detection/confirmation. The studies are heterogeneous, because they may entail different sets of accessible genes and these accessibility rates (γ_s) are study specific, and also there may be different false negative measurement errors (β_s) involved in each study due to individual experimental environment. (We had considered a single parameter γ and β, but saw substantial improvements when we allow the extra flexibility.) From study to study the data are not independent, owing to gene specific factors I_g and T_g, which get marginalized in our likelihood computation.

To further explain the model structure, the curious $G_{4,1}$ term and related terms enter because of our knockdown model. We suppose that each targeting or off-targeting event is associated with a uniformly distributed variable on $(0, 1)$. For on-targets, we suppose that four hits reduce the expression of the target such that the amount left over equals the product of the four uniforms, and if this amount is less than ω, we would see an effect, in the absence of measurement error. This happens with probability $G_{4,1}(-\log\omega)$. We further assume that off-target events hit separate genes, and act in a parallel fashion, so that a phenotype effect occurs if any of the mRNA levels is reduced below ω; this happens with probability $1-(1-\omega)^t$ when there are t off-targeting events. By modeling this way we allow that multiple on-target hits are more effective than the same number of dispersed off-target hits (Figure S1).

The 81 multi-study pattern probabilities $\{P_\pi\}$ (and thus the log-likelihood $\mathcal{L}(\psi)$) in (1) are obtained as a function of the 12 system-level parameters ψ by summing out the discrete-valued latent variables. Considering among-gene independence, we focus on a single gene, and sum out values of the involvement indicator I_g, the four accessibility indicators $A_{g,s}$, and the off-target counts T_g, the four $T_{g,s}$, and the $\{V_{g,s,k}\}_{k=1}^4$ for each study. (We model $T_{g,s}$'s as subsets of a common T_g to reflect the possibility that different studies share siRNAs.) All but the target counts are binary sums; more complicated is the elimination of the off-target counts. To investigate this calculation, write the vector $a = \{a_s\}$ and the conditional probability of data pattern π as,

$$P_\pi(i, a) = P\left(\pi | \, I_g = i, \{A_{g,s}\}_{s=1}^4 = a\right).$$

Each multi-study pattern probability P_π is computed as a summation of these $P_\pi(i, a)$ over the 2^5 values of its arguments. The trickier computation is the evaluation of each $P_\pi(i, a)$, which requires marginalization of the off-target counts.

To marginalize the off-target counts, first recognize that each pattern π is an intersection of four study-specific patterns $\pi = \bigcap_s \pi_s$. For example $\pi = 3111$ indicates that the gene is confirmed and detected in the first study and neither detected nor confirmed in any of the remaining three studies. The modeling assumptions give

$$\begin{aligned}
P_\pi(i, a) &= \sum_{t=0}^{\infty} P(T_g = t) \, P\left(\pi | I_g = i, \{A_{g,s}\}_{s=1}^4 = a, T_g = t\right) \\
&= \sum_{t=0}^{\infty} \text{Pois}(t) \prod_{s=1}^{4} P\left(\pi_s | I_g = i, A_{g,s} = a_s, T_g = t\right) \\
&= \sum_{t=0}^{\infty} \text{Pois}(t) \prod_{s=1}^{4} \sum_{u=0}^{t} B_s(t, u) \, P\left(\pi_s | I_g = i, A_{g,s} = a_s, T_{g,s} = u\right) \\
&= \sum_{t=0}^{\infty} \text{Pois}(t) \prod_{s=1}^{4} \sum_{u=0}^{t} B_s(t, u) \, Q_{s,i,a_s,u} \qquad (3)
\end{aligned}$$

where $\text{Pois}(t) = P(T_g = t) = \exp\{-K\theta\nu\}(K\theta\nu)^t/t!$ by the Poisson assumption, $B_s(t, u)$ is the Binomial mass function at u with t trials and success probability $\frac{4\gamma_s}{K}$, and where each contribution $Q_{s,i,a_s,u} = P\left(\pi_s | I_g = i, A_{g,s} = a_s, T_{g,s} = u\right)$ is computed from the stochastic model (2). Coming back to pattern $\pi = 3111$ for example, the four sub-pattern probabilities are:

$$\begin{aligned}
Q_{1,i,a_1,u} &= P\left(D_{g,1} = 1 | I_g = i, A_{g,1} = a_1, T_{g,1} = u\right) \, P\left(C_{g,1} = 1 | I_g = i, A_{g,1} = a_1, T_{g,1} = u\right) \\
Q_{2,i,a_2,u} &= P\left(D_{g,2} = 0 | I_g = i, A_{g,2} = a_2, T_{g,2} = u\right) \, P\left(C_{g,2} = 0 | I_g = i, A_{g,2} = a_2, T_{g,2} = u\right) \\
Q_{3,i,a_3,u} &= P\left(D_{g,3} = 0 | I_g = i, A_{g,3} = a_3, T_{g,3} = u\right) \, P\left(C_{g,3} = 0 | I_g = i, A_{g,3} = a_3, T_{g,3} = u\right) \\
Q_{4,i,a_4,u} &= P\left(D_{g,4} = 0 | I_g = i, A_{g,4} = a_4, T_{g,4} = u\right) \, P\left(C_{g,4} = 0 | I_g = i, A_{g,4} = a_4, T_{g,4} = u\right).
\end{aligned}$$

where

$$P\left(C_{g,s} = 1 | I_g = i, A_{g,s} = a_s, T_{g,s} = u\right) = P\left(\sum_{k=1}^{4} C_{g,s,k} \geq 2 \,\middle|\, I_g = i, A_{g,s} = a_s, T_{g,s} = u\right)$$

We make the simplifying approximation that $C_{g,s,k}$ are conditionally independent (and thus $C_{g,s}$ is governed by Binomial masses), though in fact they have some negative dependence attributable to the divvying up the off-target count $T_{g,s}$ among the four separate assays.

A key to simplifying the computation further is to recognize that with respect to the count variable u, each $P(D_{g,s}|I_g, A_{g,s}, T_{g,s})$ is a polynomial of $\xi_1 = 1-\omega$, and each $P(C_{g,s}|I_g, A_{g,s}, T_{g,s})$ is a polynomial of $\xi_2 = 1-\frac{\omega}{4}$. Thus, each $Q_{s,i,a_s,u}$ is a bivariate polynomial in ξ_1 and ξ_2, of degree at most u and $4u$ respectively. By careful book-keeping, we identify coefficients $\{b_{s,p,q}\}$ (depending on system parameters ψ and the pattern π) such that

$$Q_{s,i,a_s,u} = \sum_{p=0}^{1}\sum_{q=0}^{4} b_{s,p,q}\ (\xi_1^p\,\xi_2^q)^u$$

Thus the inner factor of (3)

$$\begin{aligned}
\sum_{u=0}^{t} B_s(t,u)\ Q_{s,i,a_s,u} &= \sum_{u=0}^{t} B_s(t,u)\ \sum_{j=0}^{8} b_{s,j}\ \xi^{uj} \\
&= \sum_{p=0}^{1}\sum_{q=0}^{4} b_{s,p,q} \sum_{u=0}^{t} (\xi_1^p\,\xi_2^q)^u\ B_s(t,u) \\
&= \sum_{p=0}^{1}\sum_{q=0}^{4} b_{s,p,q} \left(1 - \frac{4\gamma_s}{K} + \frac{4\gamma_s}{K}\xi_1^p\,\xi_2^q\right)^t \\
&= \sum_{p=0}^{1}\sum_{q=0}^{4} b_{s,p,q} {e_{s,p,q}}^t,
\end{aligned}$$

with the second-last line obtained from the moment generating function of a Binomial variable, and with $e_{s,p,q} = 1 - \frac{4\gamma_s}{K} + \frac{4\gamma_s}{K}\xi_1^p\,\xi_2^q$. Incorporating this back into (3), we obtain for the conditional probability of a pattern given accessibility and involvement:

$$\begin{aligned}
P_\pi(i,a) &= \sum_{t=0}^{\infty} \mathrm{Pois}(t) \prod_{s=1}^{4}\sum_{p=0}^{1}\sum_{q=0}^{4} b_{s,p,q} {e_{s,p,q}}^t \\
&= \sum_{t=0}^{\infty} \mathrm{Pois}(t) \sum_{p_1=0}^{1}\sum_{p_2=0}^{1}\sum_{p_3=0}^{1}\sum_{p_4=0}^{1}\sum_{q_1=0}^{4}\sum_{q_2=0}^{4}\sum_{q_3=0}^{4}\sum_{q_4=0}^{4} \left(\prod_{s=1}^{4} b_{s,p_s,q_s}\right)\left(\prod_{s=1}^{4} e_{s,p_s,q_s}\right)^t \\
&= \sum_{p_1=0}^{1}\sum_{p_2=0}^{1}\sum_{p_3=0}^{1}\sum_{p_4=0}^{1}\sum_{q_1=0}^{4}\sum_{q_2=0}^{4}\sum_{q_3=0}^{4}\sum_{q_4=0}^{4} \left(\prod_{s=1}^{4} b_{s,p_s,q_s}\right)\sum_{t=0}^{\infty} \mathrm{Pois}(t)\left(\prod_{s=1}^{4} e_{s,p_s,q_s}\right)^t \\
&= \sum_{p_1=0}^{1}\sum_{p_2=0}^{1}\sum_{p_3=0}^{1}\sum_{p_4=0}^{1}\sum_{q_1=0}^{4}\sum_{q_2=0}^{4}\sum_{q_3=0}^{4}\sum_{q_4=0}^{4} \left(\prod_{s=1}^{4} b_{s,p_s,q_s}\right)\exp\left\{K\theta\nu\left[\left(\prod_{s=1}^{4} e_{s,p_s,q_s}\right)-1\right]\right\}.
\end{aligned}$$

s where the last line comes from the moment generating function of a Poisson distribution. Finally, the pattern probability P_π is obtained by summing over the 2^5 states of i and a, as indicated previously. This provides a route to computing all 81 multi-study pattern probabilities required for likelihood evaluation.

3 Inference computations

3.1 Likelihood evaluation and maximization

Based on formulas for pattern probabilities $\{P_\pi\}$ the log-likelihood (1) was available numerically. We used some convenient facilities in the R system, including the polynom package, to organize the

rather complex sums (R Core Development Team, 2011, version 2.13.1; Venables *et al.*, 2009). To maximize the log-likelihood, we used the R function nlminb. We wrote a project specific R package, metaflu, to contain the data and main likelihood-based computations.

3.2 Posterior computation

The Metropolis-Hastings method was used to construct a Markov chain to simulate the joint posterior density

$$P(\psi|\text{data}) \propto \exp \mathcal{L}(\psi).$$

Thus, the posterior is proportional to the likelihood times a flat prior. Importantly, we did not run MCMC over the high-dimensional space including latent variables, because we were able to solve these analytically. Our sampler produced a sequence $\psi^1, \psi^2, \ldots, \psi^B$ of parameter vectors according to standard Metropolis-Hastings updating rules (e.g., Robert and Cassella, 1999, page 231.)

We systematically scanned the 12 parameter values, and used a base set of local move types that modified one parameter at a time. We call this sampler `local`. The base proposal distribution for ν was exponential, with mean at the fixed value 1/50. All other parameters resided in $(0, 1)$, and for each we used a uniform window proposal; window length 0.025 gave acceptance rates in the range 28% to 70%. Concerned about possible poor mixing caused by posterior correlation between θ and ν, we included a joint update involving $(\theta, \nu) \to (\theta c, \nu/c)$ for a Gamma-distributed multiplier c (shape, rate both 50, so mean 1).

Extensive numerical experiments indicated that from random starting points `local` would converge to one of two distinct modes. One of these modes was degenerate from the biological and technical perspective, corresponding to implausible parameter settings: involvement θ and error rates α and β_s all very close to unity. To address the lack of mixing between the degenerate and main modes, we modified the MCMC by adding to the local move types a global move type using an 'indpendence' sampler, wherein the proposed state does not depend on the current state. Specifically the proposal was an equal mixture of two 12-dimensional Gaussian distributions, with means and covariance matrices designed to match the structure of the evident posterior modes. Repeated runs of this enhanced sampler (called `global`) from random starts (uniform for rate parameters, standard exponential for ν) showed very good mixing properties (Results in Supplementary Text S2).

To address the implausibility of the degenerate posterior mode (in which θ and the error rates α and β_s were all near unity), we post-processed the MCMC output through a prior constraint: namely, we retained sampled parameters only if all θ, α, and all β_s were less than 0.8. Thus, posterior inference was based upon a flat prior, but subject to a plausibility constraint. We used marginal posterior means to estimate the parameters and the equi-tail percentile method to obtain confidence (equivalently *credible*) intervals.

3.3 Posterior distribution of N

The total number of influenza-involved genes is $N = \sum I_g$. By our approach we have marginalized the involvement indicators, and so the posterior of N needs to be obtained through further post-processing of the MCMC output. We estimate N by $G \cdot \hat{\theta}$ where $\hat{\theta}$ is the mean of posterior distribution of θ from MCMC. The posterior distribution of N is approximated as follows. For

$n = 0, 1, \ldots, G$,

$$
\begin{aligned}
P(N = n|\text{data}) &= \int P(N = n|\psi, \text{data}) p(\psi|\text{data})\, d\psi \\
&\approx \frac{1}{B} \sum_{b=1}^{B} P(N = n|\psi^b, \text{data}) \qquad (4)
\end{aligned}
$$

where $\{\psi^b\}$ constitute the MCMC output. A priori, N given ψ is distributed Binomial(G, θ), but in conditioning on the data we have a different distribution for N, even with ψ in hand. Being a sum of independent but differently-distributed Bernoulli trials, N has a Poisson Binomial distribution Thomas and Taub, 1982). For example, the one gene that is confirmed by all 4 studies is more likely to be truly involved than a gene confirmed just once. Thomas and Taub's recursion method is applied to evaluate the probability mass of N at each sampled parameter setting ψ^b.

3.4 Error rate inference

Depending on the reference set of genes, there are different ways to measure false positive and false negative error rate. In any case, we are thinking of errors in a single study, s, and define four rates

$$
\begin{aligned}
\text{FDR}(\psi) &= P\left(I_g = 0 |\, D_{g,s} = C_{g,s} = 1\right) \\
\text{FNDR}(\psi) &= P\left(I_g = 1 |\, D_{g,s} \times C_{g,s} = 0\right) \\
\text{FP}(\psi) &= P\left(D_{g,s} = C_{g,s} = 1 |\, I_g = 0\right) \\
\text{FN}(\psi) &= P\left(D_{g,s} \times C_{g,s} = 0 |\, I_g = 1\right).
\end{aligned}
$$

Respectively, these are rates of false discovery (FDR), false nondiscovery (FDNR), false positive (FP), and false negative (FN), and they all depend on the vector of system-level parameters ψ. These rates depend on probabilities in the proposed models, and are marginal to latent variables recording accessibility and off-target counts. Specifically, $P(C_{g,s} = c, D_{g,1} = d|I_g = i)$ is a summation of $Q_{s,i,a_s,u}$ over values of $A_{g,s}$ and $T_{g,s}$, as presented in Section 2 of this supplement. That covers FP and FN; for FDR and FNDR, observe that

$$
\begin{aligned}
\text{FDR}(\psi) &= \frac{P(D_{g,s} = C_{g,s} = 1|I_g = 0)\, P(I_g = 0)}{P(D_{g,s} = C_{g,s} = 1|I_g = 0)\, P(I_g = 0) + P(D_{g,s} = C_{g,s} = 1|I_g = 1)\, P(I_g = 1)} \\
&= \frac{\text{FP}(\psi)\,(1-\theta)}{\text{FP}(\psi)\,(1-\theta) + (1 - \text{FN}(\psi))\,\theta} \\
\text{FNDR}(\psi) &= \frac{[1 - P(D_{g,s} = C_{g,s} = 1|I_g = 1)]\, P(I_g = 1)}{[1 - P(D_{g,s} = C_{g,s} = 1|I_g = 1)]\, P(I_g = 1) + [1 - P(D_{g,s} = C_{g,s} = 1|I_g = 0)]\, P(I_g = 0)} \\
&= \frac{\text{FN}(\psi)\,\theta}{\text{FN}(\psi)\,\theta + (1 - \text{FP}(\psi))\,(1-\theta)}.
\end{aligned}
$$

Point estimates of error rates were obtained by plugging in an estimate $\hat{\psi}$ of system parameters, using the DL-1 study as a reference. Bayesian confidence sets were obtained by percentiling error rate values computed across MCMC samples $\{\psi^b\}$.

4 Code

The metaflu package and associated R source code is available at
`http://www.stat.wisc.edu/~newton/metaflu`.

References

1. Basu, S and Ebrahimi, N (2001). Bayesian capture-recapture methods for error detection and estimation of population size: heterogeneity and dependence. *Biometrika*, 88, 269-279.

2. Craig, BA, Newton, BA, Garrott, RA, Reynolds, JE, and Wilcox, JR (1997). Analysis of aerial survey data on Florida manatee using Markov chain Monte Carlo. *Biometrics*, 53, 129-146.

3. Fisher, RA, Corbet, AS, and Williams, CB (1943). The relation between the number of species seen and the number of individuals in a random sample of an animal population. *Journal of Animal Ecology*, 12, 42-58.

4. R Development Core Team (2011). *R: A language and environment for statistical computing.* R Foundation for Statistical Computing, Vienna, Austria. ISBN 3-900051-07-0, URL `http://www.R-project.org/`.

5. Raftery, AE (1988). Inference for the binomial N parameter: A hierarchical Bayes approach. *Biometrika*, 75, 223-228.

6. Robert, C.P. and Casella, G (1999). *Monte Carlo Statistical Methods*, New York, Springer.

7. Thomas, M.A. and Taub, A.E. (1982). Calculating binomial probabilities when the trial probabilities are unequal. *Journal of Statistical Computation and Simulation*, 14, 125-131.

8. Venables, B, Hornik, K, and Maechler, M (2009). polynom: A collection of functions to implement a class for univariate polynomial manipulations. R package version 1.3-6.

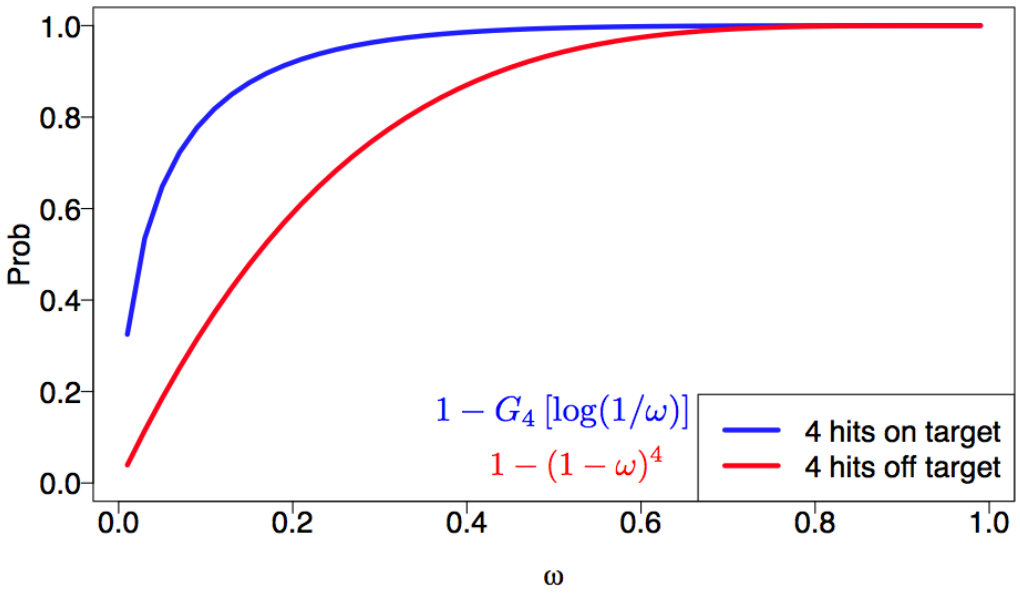

Figure S1: Knockdown model properties. Four on target hits are more effective at producing an observed effect than four off-target hits. Shown is the case of no measurement error, as a function of the threshold for an effect.
