# Supplementary material for: Limited Agreement of Independent RNAi Screens for Virus-Required Host Genes Owes More to False-Negative than False-Positive Factors
Source: PLoS Comput Biol. 2013 Sep 19;9(9):e1003235. doi: 10.1371/journal.pcbi.1003235 (PMC3777922; doi:10.1371/journal.pcbi.1003235)
Supplement: Text S2 — Statistical model validation, inference, and prediction. (PDF) [file pcbi.1003235.s003.pdf]

Supplementary Material

**Text S2:**	Statistical Model Validation, Inference, and Prediction
**Main paper by:**	Hao, L, He, Q, Craven, M, Newton, MA, and Ahlquist, P
**Date:**	May 21, 2013
**Contact:**	Michael A. Newton, `newton@stat.wisc.edu`

1 Outline

Regarding the statistical model presented in the main paper, the present document provides additional details on model validation, inference summaries, and prediction results. We employed a variety of computer experiments for related purposes: (1) to test that our code was calculating what we intented it to calculate, (2) to assess goodness-of-fit of the proposed model, (3) to check the robustness of conclusions to various model assumptions, and (4) to obtain and evaluate model-based predictions, including an application to HIV RNAi. This supplement also contains details of the model fit to the influenza data.

2 Diagnostics

2.1 Consistency checks

The code base was relatively complex and required substantial testing. Among the basic checks was a useful consistency check. In parametric models, the MLE is known to be consistent. Hence, if we simulated sufficiently many draws from the 81-cell multinomial (i.e., sufficiently many genes), the computed MLE would need to be close to the generating parameter vector. In one test, we increased the genome size from 22000 to 10^6 or 10^7, generated the 81-pattern counts from various parameter settings, and ran the optimization code to estimate the underlying parameters. Parameters values were accurately recovered. Occasionally, `nlminb` would get stuck in local modes and multiple restarts we required from independent random initial values. (See the `nlogpost4` function of the `metaflu` R package cited at the end of this supplement.)

2.2 Predictive checks

Model development was characterized by a series of tests of the model's ability to recapitulate features in the data, as well as to represent presumed structures in RNAi data. (i.e. we did not start with a model as complex as the one finally presented here!) We employed forward simulation to generate synthetic multi-study data: i.e., we repeatedly simulated latent involvement indicators, accessibility indicators, and off-target counts, followed by detection and confirmation indicators, after fixing the system-level parameters at certain values. based on various predictive checks. Table S2 shows observed counts compared to estimates from 1000 multi-study simulations at the fitted parameter values. Discrepancies in the generally good fit are attributable to Monte Carlo error and also the approximation error originating in our treatment of the confirmation-screen data. Figure S3 is a marginal histogram, from this same simulation, showing the number of confirmed genes from across the multiple studies (we observed 614 and the fitted predictive distribution covers this value well.) Figure S2 reveals another characteristic of the observed data that is well approximated by the fitted model; namely, the overall numbers of detected and confirmed genes per study. These three basic checks indicate a good model fit for the statistics considered. We note that a version of the model which did not allow parameter heterogeneity among studies showed lack of fit in the detection/confirmation plot.

2.3 Leave-one-study-out diagnostics

As further validation of our model-based approach, we checked how well it estimated parameters when data from three studies were used to fit the model. This cross-validation exercise provides some assessment of the stability of inference. With four studies there are four leave-one-out cases; for each we developed inference computations for model fitting. Some care was required to reduce from the table of 81 (3^4) four-study patterns to tables of 27 (3^3) three-study detection/confirmation patterns.

Following the posterior prediction strategy described below (Section 4), we simulated counts of how many novel genes would be confirmed by a fourth study given data from three studies, and we compared these predictions to available data (Table S3). In all four cases, the count predicted from tri-study training data matched well to the observed test data that had been left out.

Parameter estimates from the four leave-one-out cases are shown in Table S4. Reflecting inferential stability, these estimates are very similar to results based on all four studies. Sizes of detection and confirmation patterns influence the results. For example, $A549DE$ has the most overlap with other studies in confirmed genes, when it is excluded, the estimated θ value is most affected.

2.4 Robustness checks

In developing model-based inference for factors affecting multi-study RNAi data, we had formulated a range of models prior to the final model presented in the main paper. We settled on the final model because it exhibited a goodness of fit, it made plausible predictions, and it captured what we could formulate about the key systematic sources of variation. Earlier models (not shown) failed on one or more of these criteria. We report here one additional test of the final model assumptions.

Our main computations treated the number T_g of influenza-involved off-targets of each first-round siRNA pool as Poisson distributed (with mean of $K\theta\nu$ to account for the pool size, the involvement rate, and the overall rate of off targeting). A first-principles argument supports this assumption, and experience suggests that the impact of violations in this assumption on other inferences is probably minimal. However, the limited data on off-target rates suggests variation in T_g that is more extensive than the Poisson (Kulkarni, *et al.* 2006). To check the robustness of our Poisson-based approach, we investigated replacing the Poisson distribution with the Negative Binomial distribution to allow potential overdispersion. Specifically, for a Gamma distributed random variable C, with both shape and rate parameters equal to κ (and thus mean 1), we considered:

$$T_g|[C = c] \quad \sim \quad \text{Poisson}(K\theta\nu c),$$

which implies

$$T_g \quad \sim \quad \text{Negative Binomial}\left(\kappa, \frac{K\theta\nu}{K\theta\nu + \kappa}\right)$$

and parameterized so the mean continues to be $K\theta\nu$. Small $\kappa > 0$ corresponds to substantial overdispersion, while $\kappa \longrightarrow \infty$ recapitulates the Poisson model. Complexity of the multi-study pattern probabilities put a full analysis of the Negative Binomial model beyond our reach, though we were able to obtain pattern probabilities in several boundary cases. As the siRNA pool size K gets large, off-target counts $T_{g,s}$ from different studies become independent, and thus data from the separate studies become conditionally independent given the involvement indicators. In this limiting case,

$$T_{g,s} \sim \text{Negative Binomial}\left(\kappa, \frac{4\theta\nu\gamma_s}{4\theta\nu\gamma_s + \kappa}\right)$$

and the simplifications arising from independent studies enabled us to compute all pattern probabilities for likelihood analysis. In this independent-study case, we recomputed the maximum likelihood

parameter values over a grid of κ values in $(0, 1000)$. We found very little dependendence of estimates on the value of κ. To link back to the actual case (among-study dependence, and small K), we retained the Poisson model but varied K over the range $(4, 1000)$. Again we saw very little dependence of the MLEs on the value of K. This lack of sensitivity to K and κ may be due to the data favoring very small mean off-target rate ν; with small ν, the likelihood surface is relatively flat over the domains of K and κ.

As a further investigation of the off-target rate, we considered a range of values ν (on a grid) and at each one profiled the remaining parameters by maximum (profile) likelihood. Results shown in the main text show that increasing ν does not explain the data well (decreasing likelihood fit), and further that a reason for this is the constrained model's inability to explain the relatively high confirmation rate. Figure S9 presents another view of this lack-of-fit, in the spirit of the goodness-of-fit plot in Figure S2.

3 Inference summaries

Working on the log scale for ν and the logit scale for all probabilities, the optimization code was initiated at the zero vector to compute maximum likelihood estimates (MLEs). Numerical experiments showed insensitivity to a range of starting configurations.

For the MCMC computation, the `local` sampler was initiated at the MLE parameter vector, run for length 250000 scans, and subsampled every 100 scans for final output. Acceptance rates of parameters was between 29% and 70%. Trace plots (Figure S4) and autocorrelation plots (Figure S5) indicated good mixing properties within the dominant posterior mode.. The total number of involved genes is a parameter whose distribution is induced by the distribution of other parameters; its posterior was calculated after MCMC (Figure S8).

To assure that posterior summaries were not sensitive to starting position of the Markov chain, we investigated sample behavior from random initial conditions, and noted that `local` could be drawn into and trapped within a degenerate posterior mode near the boundary of the parameter space where θ, α, and all β_s were near unity. We thus applied a modifed sampler, called `global`, which incorprated an independence-sampler move type, and we filtered the output to retain parameters only if θ, α, and all β_s were less than 80% (Supplementary Text S1). Repeated runs of `global` from random initial conditions (uniform on all rate parameters, standard exponential on ν) demonstrates posterior convergence, insensitivity to initial condition, and equivalence with the output of `local` run from the MLE (Figure S6).

4 Predicting outcomes in future siRNA studies

The model-based approach provides a mechanism for predicting outcomes of further siRNA studies. We pursued posterior predictive simulation in which parameter draws from the MCMC output were used to seed forward simulation of further siRNA studies (we mixed over the four posteriors for study-specific error rates to incorporate parameter settings for these hypothetical future studies.) Specifically, for each of 2000 posterior draws, we simulated a future trajectory of up to 50 future studies. Each trajectory represented a state of nature, and so corresponded to a single draw of involvement indicators $\{I_g\}$ and off-target numbers $\{T_g\}$. Along each trajectory, we sampled accessibilities and study-specific off-target numbers $T_{g,s}$ at each step, and we generated detections and confirmations. We kept track of how many novel genes were confirmed along the way. A subtlety of the computation was making it *posterior* predictive. There were up to 81 different kinds of trajectories, depending on the four-study data on a given gene from the existing data; and each kind corresponded to different involvement and off-target inferences.

5 Application to HIV studies

As a further validation exercise we checked how the model-based approach worked on an independent collection of three RNAi experiments from the study of HIV (Brass *et al.* 2008; Zhou *et al.* 2008; Konig *et al.* 2008). These studies used similar two-stage designs and experimental procedures to the four influenza studies, and so we organized the mult-HIV-study data into a table holding patterns of detection/confirmation across the three studies and we fit the proposed model to these data.

Because only one of the three HIV studies reported data on both primary and secondary screens, we did not have access to all $3^3 = 27$ counts, and were forced to use a reduced set of 12 pattern counts (see Table S5). Recall that in original patterns, digits $0, 1, 2$ refer to respectively detection and confirmation status $\{D_{g,s} = C_{g,s} = 0\}, \{D_{g,s} = 1, C_{g,s} = 0\}, \{D_{g,s} = C_{g,s} = 1\}$. For instance, that a gene has pattern 201 means that it is detected and confirmed by study 1, not detected nor confirmed by study 2, and detected but not confirmed by study 3. Suppose we only have detection and confirmation data from study 3 in HIV meta analysis, then we are able to identify detection and confirmation status 0, 1, or 2 for only study 3. For the other 2 studies, we are only able to identity if the status is 2 or not, but not able to differentiate 0 from 1. Therefore, what have previously been patterns 201 and 211 need to be collapsed into one single pattern which is collapsed pattern 9 in Table S5. Because of the limited available pattern information, we used a common false negative error β instead of 4 study specific ones for a better model fit.

Estimated parameters, number of involved genes, error rates and their 95% credible intervals are summarized in Table S6. We use acronyms to refer to the 3 studies (SCI: Brass *et al.* 2008; CHM: Zhou *et al.* 2008; CEL: Konig *et al.* 2008). Error rates are calculated based on accessibility rate estimated for CHM study.

References

1. Brass AL, Dykxhoorn DM, Benita Y, Yan N, Engelman A, Xavier RJ, Lieberman J, Elledge SJ (2008). Identification of host proteins required for HIV infection through a functional genomic screen. *Science*, 319, 921-6.

2. Konig R, Zhou Y, Elleder D, Diamond TL, Bonamy GMC, Irelan JT, Chiang C, Tu BP, De Jesus PD, Lilley CE, Seidel S, Opaluch AM, Caldwell JS, Weitzman MD, Kuhen KL, Bandyopadhyay S, Ideker T, Orth AP, Miraglia LJ, Bushman FD, Young JA, Chanda SK (2008). Global analysis of host-pathogen interactions that regulate early-stage HIV-1 replication. *Cell*, 135, 49-60.

3. Kulkarni MM, Booker M, Silver SJ, Friedman A, Hong P, Perrimon N, Mathey-Prevot B (2006). Evidence of off-target effects associated with long dsRNAs in *Drosophila melanogaster* cell-based assays. *Nature Methods*, 3, 833-838.

4. Zhou H, Xu M, Huang Q, Gates AT, Zhang XD, Castle JC, Stec E, Ferrer M, Strulovici B, Hazuda DJ, Espeseth AS (2008). Genome-scale RNAi screen for host factors required for HIV replication. *Cell Host Microbe*, 4, 495-504.

Tables and Figures: Supplementary Text S2

Table S2. Observed and fitted multi-study influenza counts.

Table S3. Cross validation, predictions for left-out studies.

Table S4. Cross validation, estimated parameters.

Table S5. HIV data, pattern structure.

Table S6. HIV data, estimated parameters.

Figure S2. Goodness-of-fit predictions for detection/confirmation counts.

Figure S3. Predicted number of genes detected in four studies.

Figure S4. MCMC trace plots, `local` sampler.

Figure S5. Autocorrelation plots for MCMC output, `local` sampler.

Figure S6. Convergence of MCMC from random starts (`global` sampler) compared to results from `local` sampler started at MLE.

Figure S7. Estimated probabilities, 81-cell table.

Figure S8. Posterior distribution of number of influenza involved genes.

Figure S9. Lack of fit when off-target rate constrained to be large.

Table S2: Comparison between the observed 81-pattern counts, their estimated values from the fitted model and the empirical estimates from 1000 simulations. Code: 0-not detected or confirmed; 1-detected not confirmed; 2-detected and confirmed.

DL-1	U2OS	A549DE	A549US	Pattern	Observation	Model Fit	Simulation Mean	Std
0	0	0	0	0	21016	20999.02	20799.02	34.35
0	0	0	1	1	71	75.8	98.84	9.87
0	0	0	2	2	179	180.96	165.38	12.27
0	0	1	0	10	106	109.12	185.22	13.8
0	0	1	1	11	0	0.61	2.66	1.63
0	0	1	2	12	6	4.06	10.62	3.2
0	0	2	0	20	126	138.24	130.39	11.51
0	0	2	1	21	2	0.86	2.44	1.6
0	0	2	2	22	18	12.16	11.77	3.53
0	1	0	0	100	113	104.34	168.22	13.07
0	1	0	1	101	0	0.56	2.32	1.53
0	1	0	2	102	1	3.71	9.4	3.13
0	1	1	0	110	0	1.24	7.29	2.78
0	1	1	1	111	0	0.01	0.13	0.36
0	1	1	2	112	0	0.08	0.62	0.78
0	1	2	0	120	2	2.83	7.37	2.68
0	1	2	1	121	0	0.02	0.13	0.36
0	1	2	2	122	0	0.25	0.67	0.82
0	2	0	0	200	111	105.26	99.19	9.88
0	2	0	1	201	1	0.65	1.96	1.39
0	2	0	2	202	3	9.26	8.93	3.03
0	2	1	0	210	2	2.36	6.41	2.56
0	2	1	1	211	0	0.02	0.1	0.32
0	2	1	2	212	0	0.21	0.6	0.8
0	2	2	0	220	3	7.09	6.91	2.5
0	2	2	1	221	0	0.04	0.13	0.36
0	2	2	2	222	3	0.63	0.62	0.84
1	0	0	0	1000	80	74.85	96.79	10.16
1	0	0	1	1001	0	0.37	0.98	1.01
1	0	0	2	1002	2	1.15	2.97	1.68
1	0	1	0	1010	0	0.58	2.47	1.61
1	0	1	1	1011	0	0.01	0.04	0.2
1	0	1	2	1012	0	0.03	0.18	0.44
1	0	2	0	1020	1	0.88	2.42	1.59
1	0	2	1	1021	0	0.01	0.04	0.2
1	0	2	2	1022	0	0.08	0.21	0.47
1	1	0	0	1100	0	0.54	2.15	1.49
1	1	0	1	1101	0	0	0.04	0.18
1	1	0	2	1102	0	0.02	0.17	0.44
1	1	1	0	1110	0	0.01	0.12	0.33
1	1	1	1	1111	0	0	0	0.04
1	1	1	2	1112	0	0	0.01	0.1
1	1	2	0	1120	0	0.02	0.14	0.36
1	1	2	1	1121	0	0	0.01	0.09
1	1	2	2	1122	0	0	0.01	0.11
1	2	0	0	1200	0	0.67	1.83	1.34
1	2	0	1	1201	0	0	0.04	0.18
1	2	0	2	1202	0	0.06	0.17	0.42
1	2	1	0	1210	0	0.02	0.1	0.32
1	2	1	1	1211	0	0	0.01	0.08
1	2	1	2	1212	0	0	0.01	0.09
1	2	2	0	1220	0	0.04	0.12	0.35
1	2	2	1	1221	0	0	0	0.07
1	2	2	2	1222	0	0	0.01	0.09
2	0	0	0	2000	127	126.22	116	10.51
2	0	0	1	2001	1	0.79	2.1	1.45
2	0	0	2	2002	2	11.09	10.34	3.16
2	0	1	0	2010	4	2.83	7.45	2.69
2	0	1	1	2011	0	0.02	0.13	0.37
2	0	1	2	2012	0	0.25	0.64	0.79
2	0	2	0	2020	6	8.49	8.12	2.88
2	0	2	1	2021	0	0.05	0.14	0.37
2	0	2	2	2022	3	0.75	0.72	0.83
2	1	0	0	2100	4	2.59	6.66	2.57
2	1	0	1	2101	0	0.02	0.12	0.35
2	1	0	2	2102	0	0.23	0.55	0.73
2	1	1	0	2110	0	0.06	0.42	0.64
2	1	1	1	2111	0	0	0.01	0.08
2	1	1	2	2112	0	0.01	0.03	0.17
2	1	2	0	2120	1	0.17	0.46	0.69
2	1	2	1	2121	0	0	0.01	0.08
2	1	2	2	2122	0	0.02	0.04	0.2
2	2	0	0	2200	2	6.46	6.16	2.56
2	2	0	1	2201	0	0.04	0.12	0.34
2	2	0	2	2202	0	0.57	0.56	0.75
2	2	1	0	2210	0	0.14	0.34	0.56
2	2	1	1	2211	0	0	0.01	0.09
2	2	1	2	2212	1	0.01	0.04	0.19
2	2	2	0	2220	2	0.44	0.43	0.67
2	2	2	1	2221	0	0	0.01	0.08
2	2	2	2	2222	1	0.04	0.05	0.22

Table S3: Predicted number of extra genes confirmed by a 4th study based on modeling the other three studies.

Leave Out	Predicted Additional	95% Prediction Interval	Observed Additional
DL-1	139	(56, 253)	136
U2OS	143	(67, 253)	114
A549DE	156	(57, 330)	131
A549US	131	(55, 240)	188

Table S4: Estimated parameters by four ways of leaving out one study.

Leave out	$\hat{\theta}$	$\hat{\gamma}$				$\hat{\omega}$	$\hat{\beta}$				$\hat{\nu}$	$\hat{\alpha}$
		DL-1	U2OS	A549DE	A549US		DL-1	U2OS	A549DE	A549US		
DL-1	0.106	-	0.367	0.350	0.111	0.896	-	0.118	0.144	0.112	0.010	0.003
U2OS	0.102	0.190	-	0.368	0.127	0.885	0.096	-	0.159	0.120	0.010	0.003
A549DE	0.192	0.203	0.422	-	0.129	0.892	0.054	0.079	-	0.066	0.013	0.003
A549US	0.115	0.121	0.380	0.337	-	0.890	0.075	0.113	0.129	-	0.013	0.003

Table S5: HIV analysis: relation between collapsed patterns and original patterns.

Collapsed Patterns	Original Patterns			
1	222			
2	221			
3	220			
4	202	212		
5	022	122		
6	200	210		
7	020	120		
8	021	121		
9	201	211		
10	002	012	102	112
11	000	010	100	110
12	001	011	101	111

Table S6: Estimated parameters, number of involved genes and error rates and their 95% credible intervals in HIV studies.

Parameter	Point Estimate	95% C.I.
$\hat{\theta}$	0.285	(0.209, 0.390)
$\hat{\alpha}$	0.002	(0.000, 0.003)
$\hat{\beta}$	0.078	(0.009, 0.154)
$\hat{\gamma} : SCI$	0.051	(0.036, 0.070)
$\hat{\gamma} : CEL$	0.055	(0.038, 0.074)
$\hat{\gamma} : CHM$	0.043	(0.030, 0.058)
$\hat{\omega}$	0.833	(0.651, 0.990)
$\hat{\nu}$	0.017	(0.000, 0.061)
Number of Involved Genes	Point Estimate	95% C.I.
N	6277	(4591, 8620)
Error Rate of CHM Study	Point Estimate	95% C.I.
FDR	0.008	(0.000, 0.032)
$FNDR$	0.278	(0.201, 0.384)
TP	0.037	(0.026, 0.051)
TN	1.000	(1.000, 1.000)

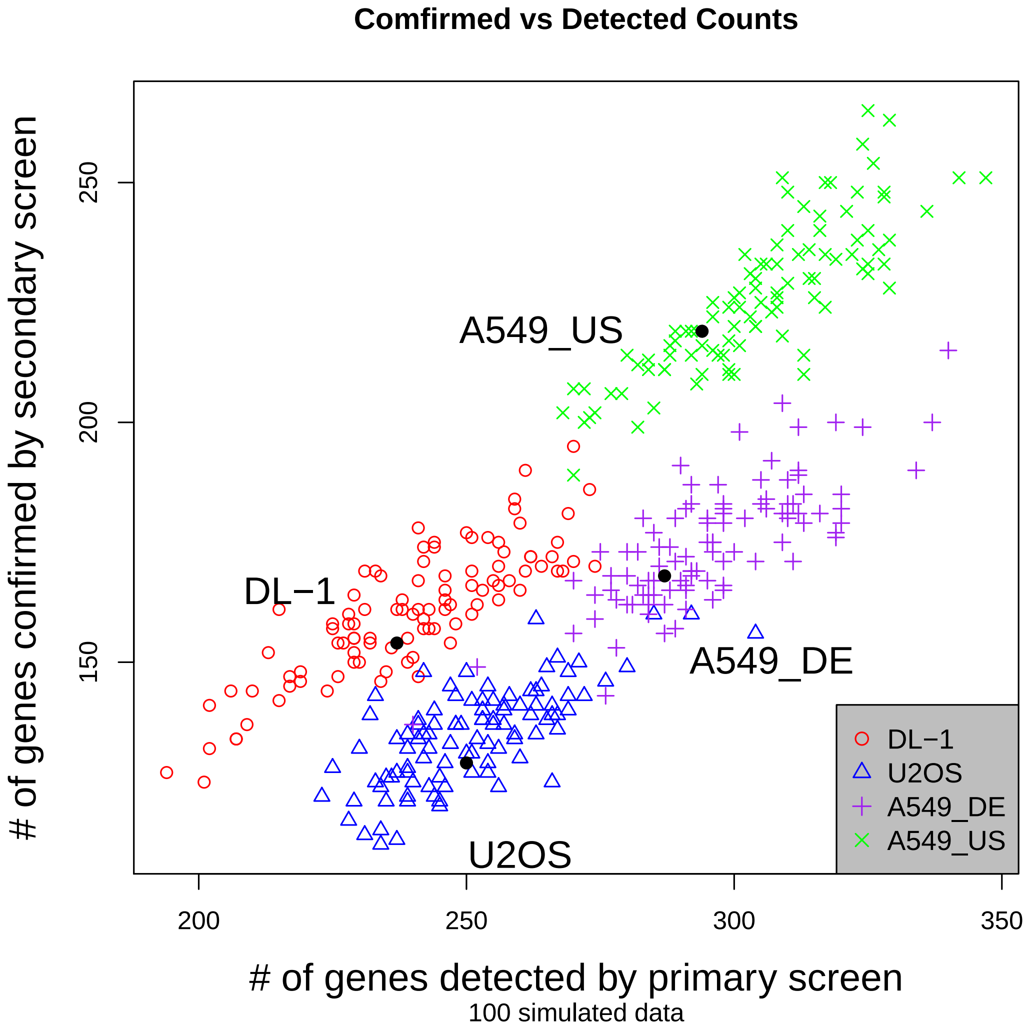

Figure S2: Observed numbers of detections/confirmations over four studies (black dots) compared to simulated values (colored symbols) from fitted model.

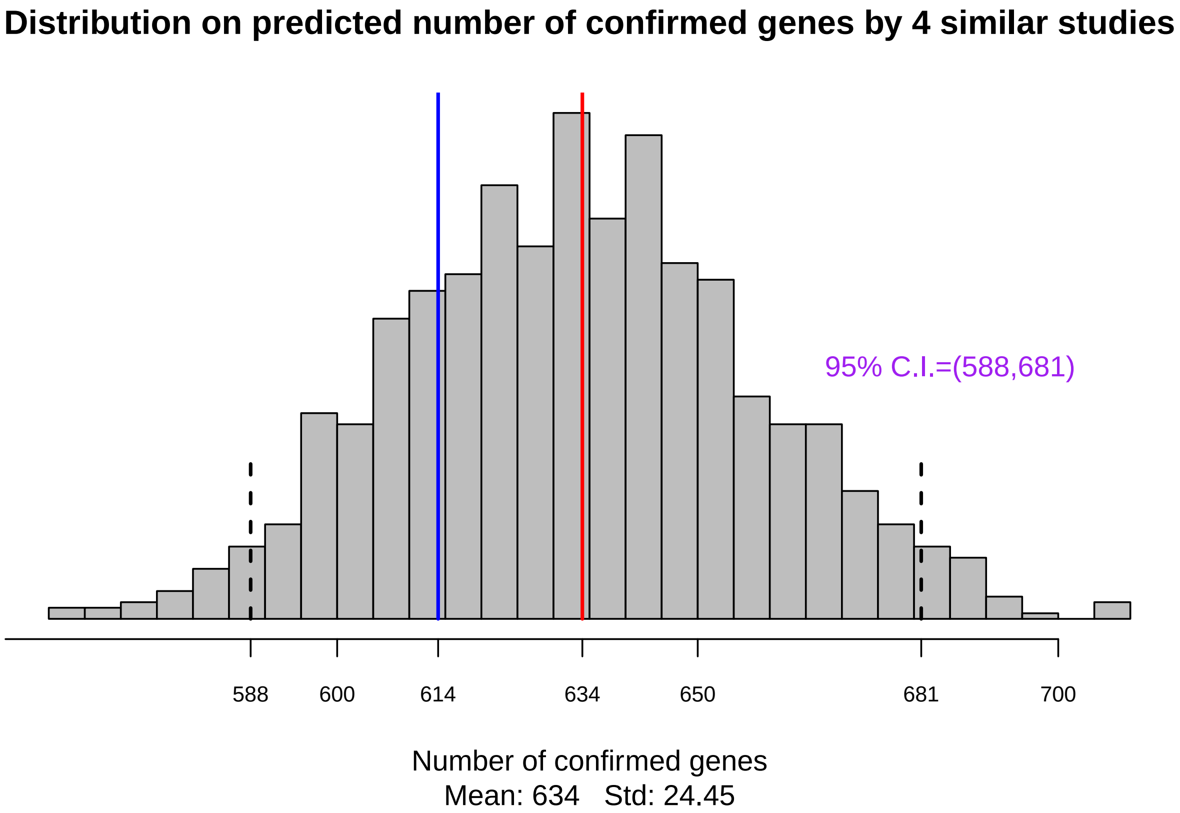

Figure S3: Goodness-of-fit simulations. Histogram of number of genes confirmed jointly by all 4 studies from 1000 simulations based on the fitted model.

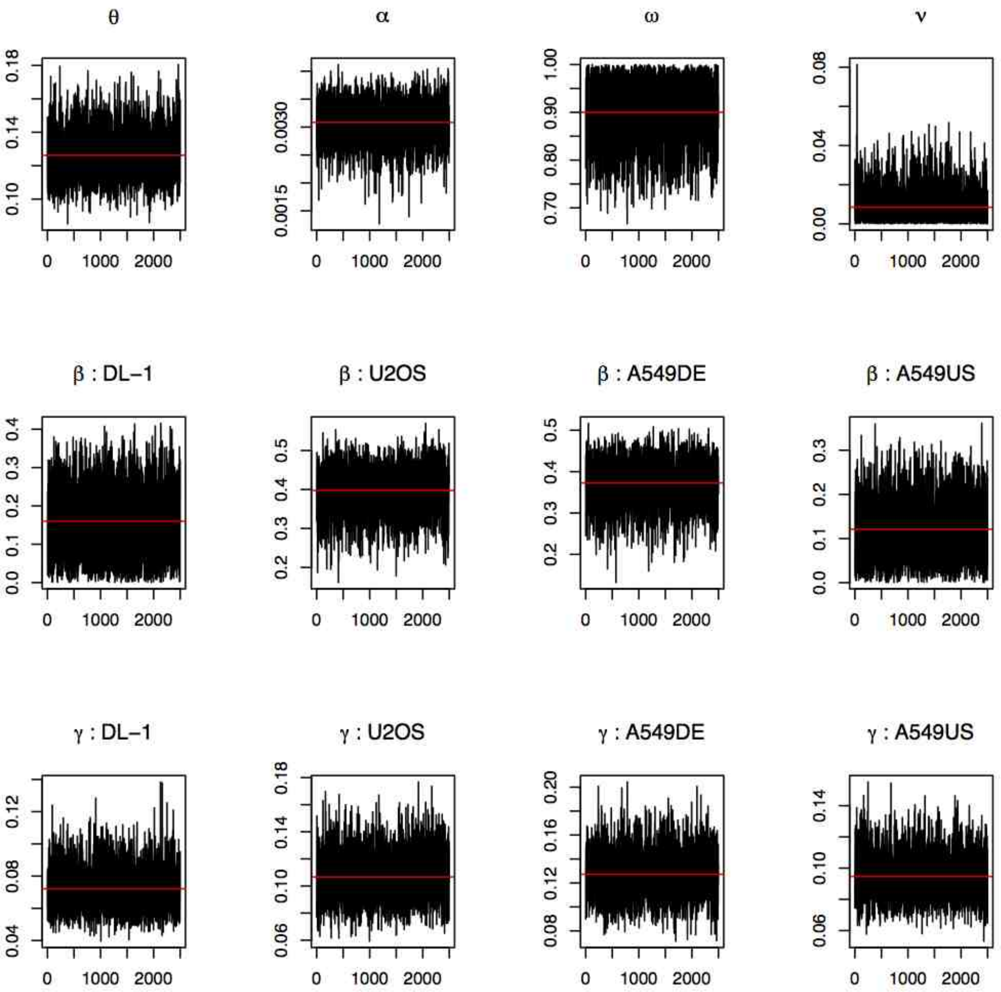

Figure S4: Trace plots parameter values from MCMC output, *local* sampler initiated at MLE.

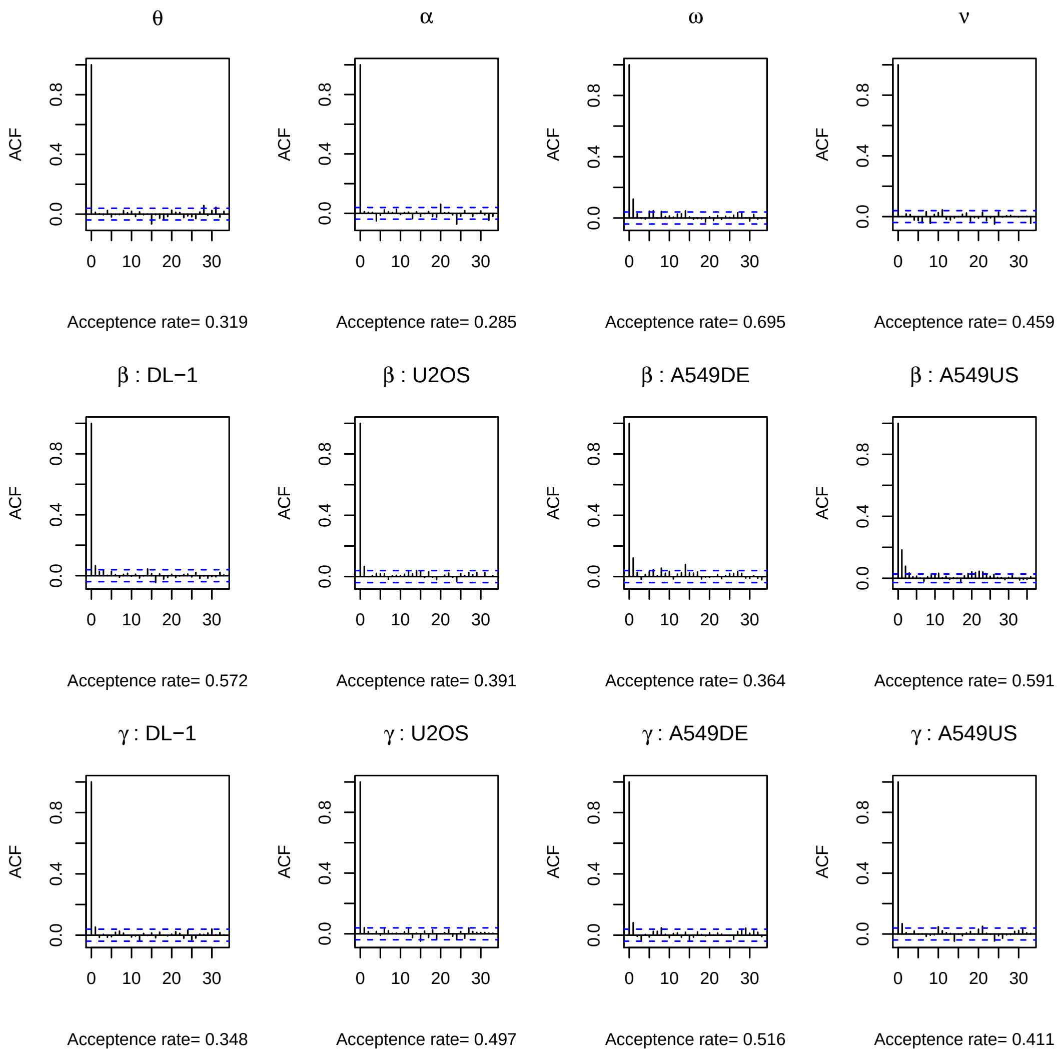

Figure S5: Autocorrelation plots of MCMC output, *local* sampler.

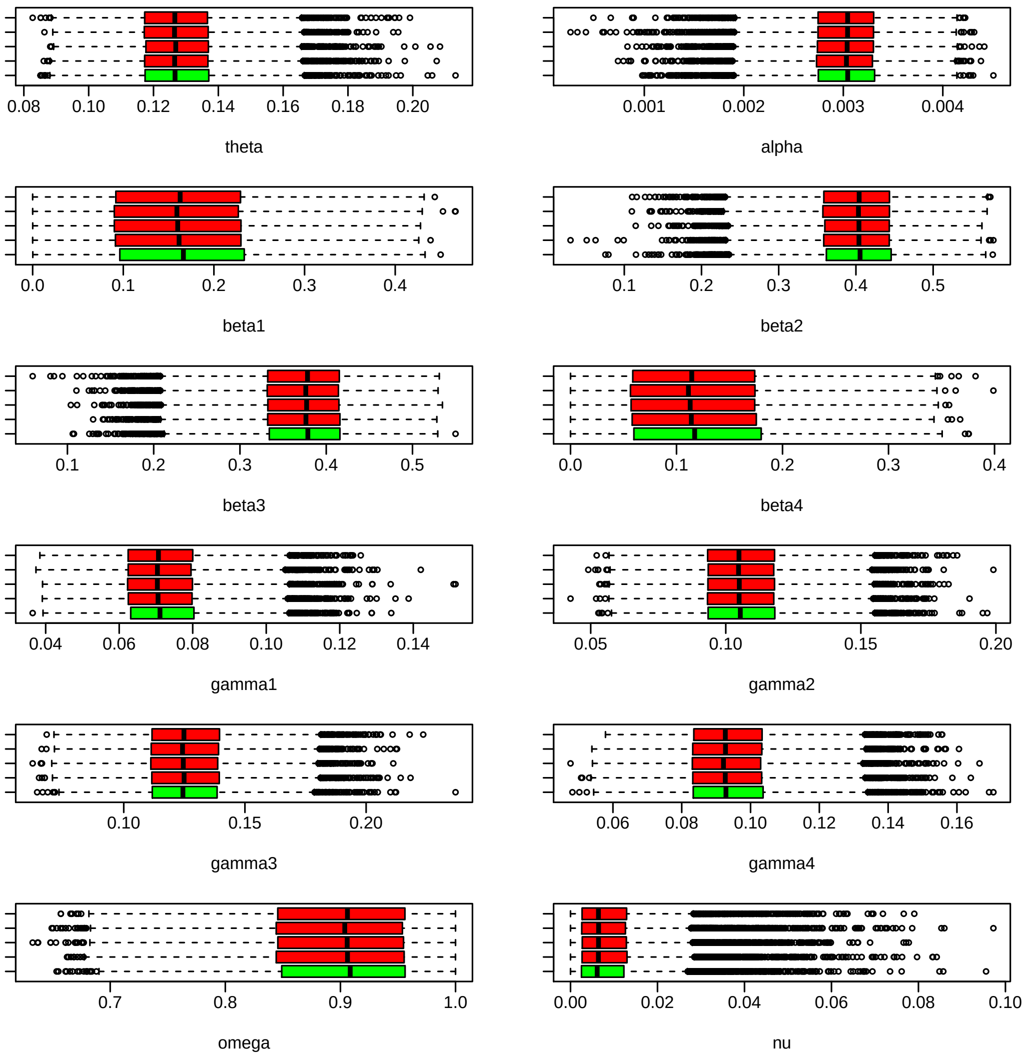

Figure S6: MCMC convergence of *global* sampler from four random starts (red) and the *local* sampler started from the MLE (green). All chains were run for 10^6 scans and subsampled every 10^2 scans.

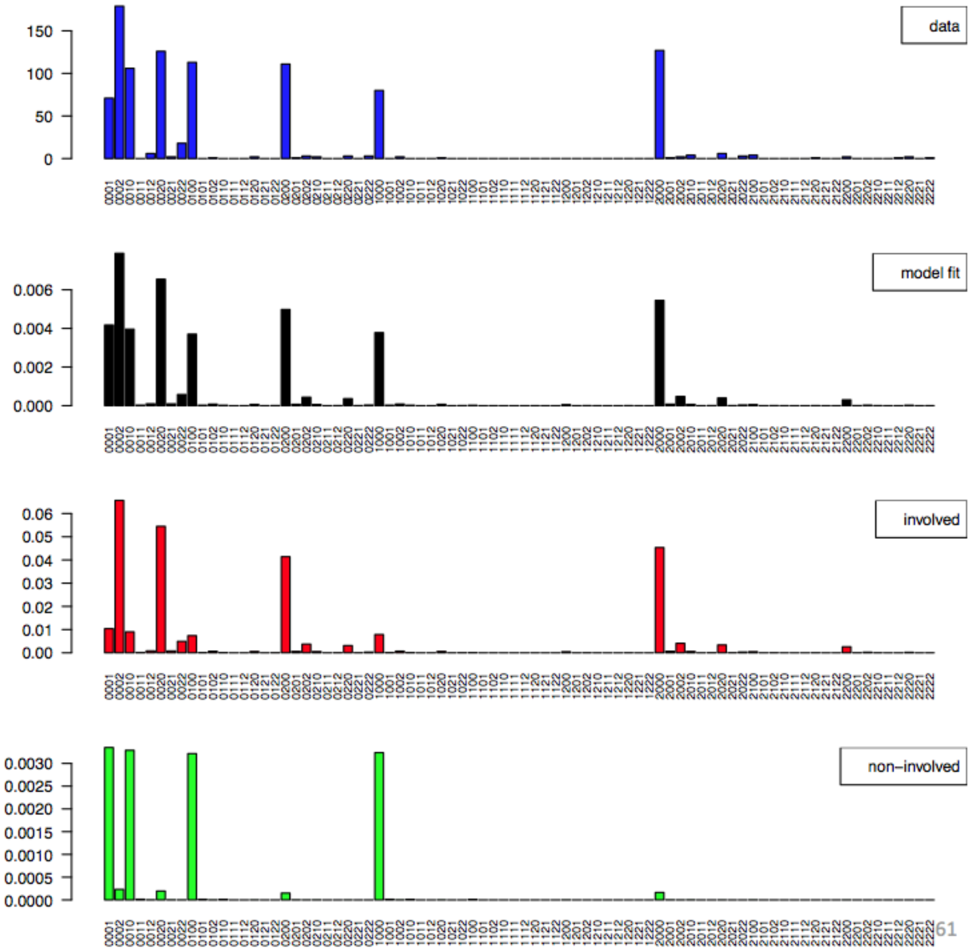

Figure S7: Data and estimated frequencies, 81 cell table. Except for the null cell (genes never detected by any study), shown are data (top panel), model fits (2nd panel), and the fit decomposed into involved and non-involved genes.

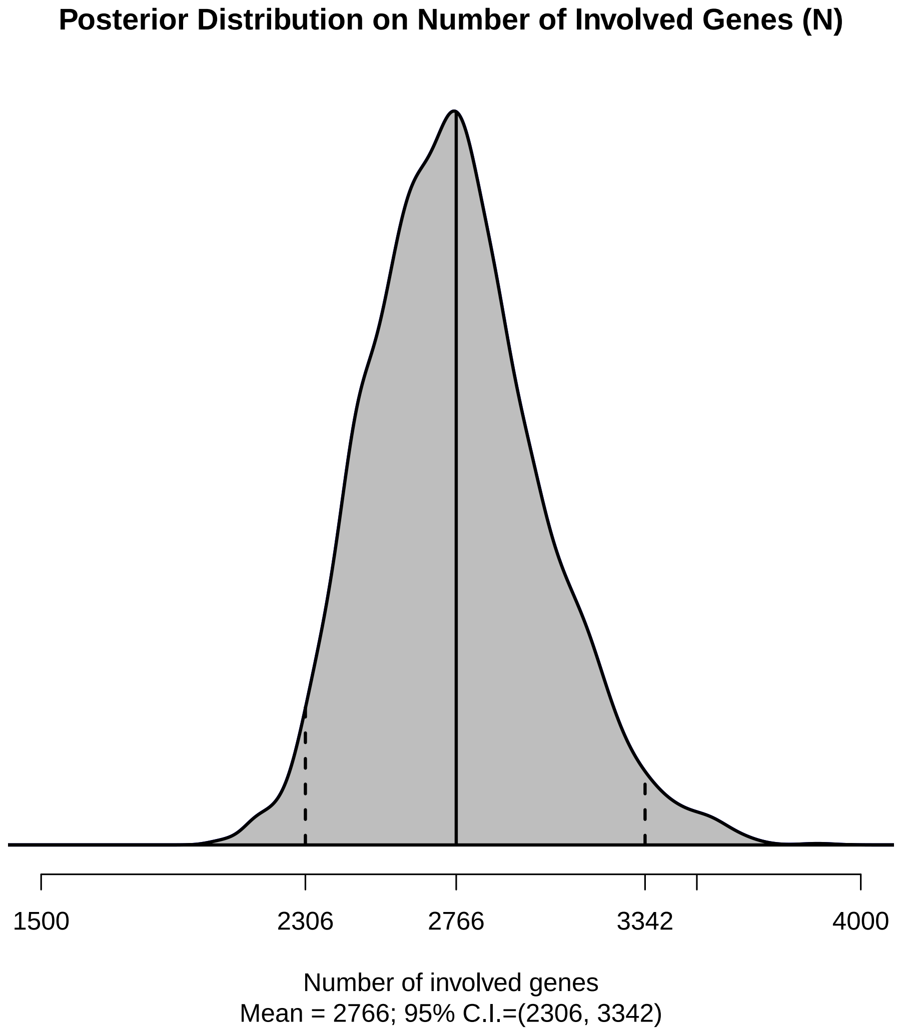

Figure S8: Posterior distribution of number N of involved genes

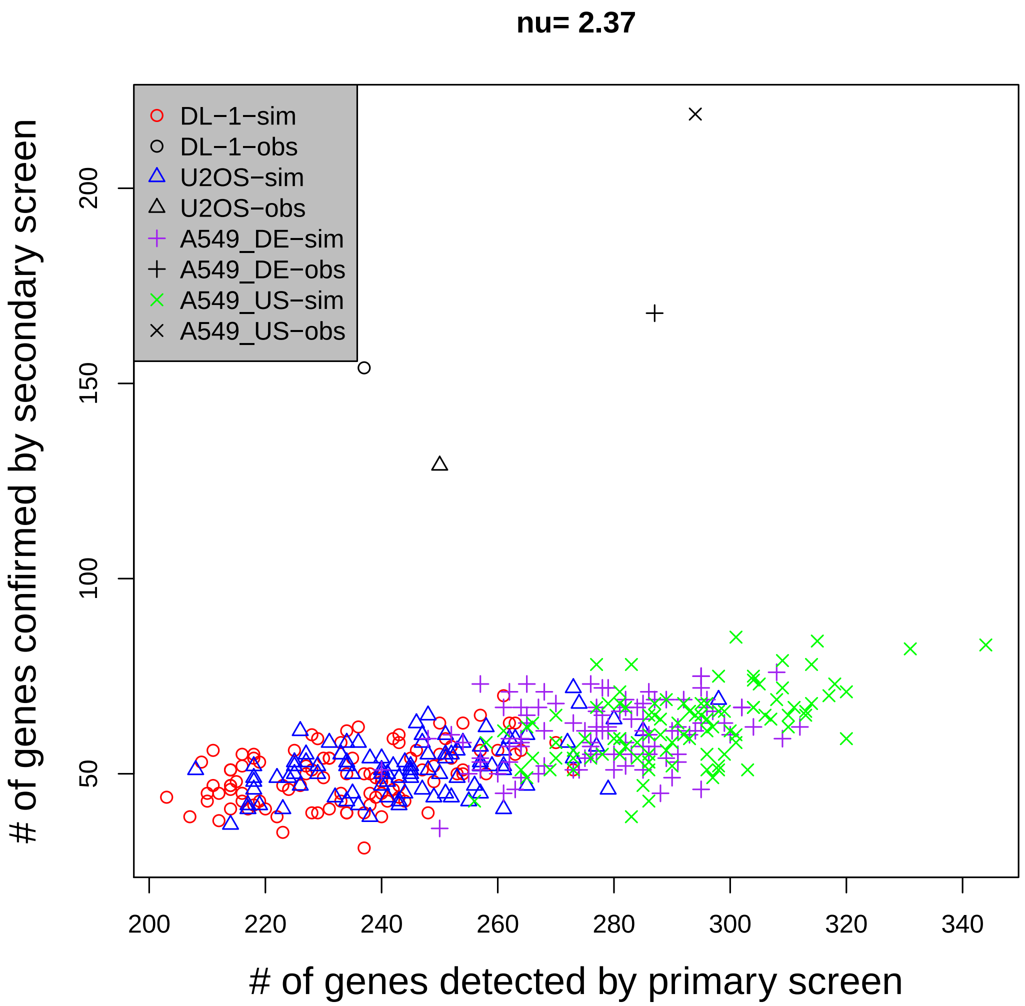

Figure S9: Lack-of-fit consequence of raising off-target rate ν. In profile computations, we fixed ν at a moderately large value, and estimated other parameters by maximum likelihood. Shown is a scatterplot revealing the constrained model's inability to explain the high confirmation rate. (Compare to Figure S2.)
